# Potential of Plant-Based Oil Processing Wastes/By-Products as an Alternative Source of Bioactive Compounds in the Food Industry

**DOI:** 10.3390/foods14152718

**Published:** 2025-08-02

**Authors:** Elifsu Nemli, Deniz Günal-Köroğlu, Resat Apak, Esra Capanoglu

**Affiliations:** 1 Department of Food Engineering, Faculty of Chemical and Metallurgical Engineering, Istanbul Technical University, 34469 Maslak, Istanbul, Turkey; elifsunemli@gmail.com (E.N.); gunald@itu.edu.tr (D.G.-K.); 2 Department of Chemistry, Division of Analytical Chemistry, Faculty of Engineering, Istanbul University, 34320 Avcılar, Istanbul, Turkey; rapak@istanbul.edu.tr; 3 Turkish Academy of Sciences (TUBA), Bayraktar Neighborhood, Vedat Dalokay St. No: 112, 06670 Çankaya, Ankara, Turkey

**Keywords:** functional food ingredients, edible oil processing waste, agro-industrial residues, antioxidant-rich, oilcake utilization, waste/by-product valorization

## Abstract

The plant-based oil industry contributes significantly to food waste/by-products in the form of underutilized biomass, including oil pomace, cake/meal, seeds, peels, wastewater, etc. These waste/by-products contain a significant quantity of nutritious and bioactive compounds (phenolics, lignans, flavonoids, dietary fiber, proteins, and essential minerals) with proven health-promoting effects. The utilization of them as natural, cost-effective, and food-grade functional ingredients in novel food formulations holds considerable potential. This review highlights the potential of waste/by-products generated during plant-based oil processing as a promising source of bioactive compounds and covers systematic research, including recent studies focusing on innovative extraction and processing techniques. It also sheds light on their promising potential for valorization as food ingredients, with a focus on specific examples of food fortification. Furthermore, the potential for value creation in the food industry is emphasized, taking into account associated challenges and limitations, as well as future perspectives. Overall, the current information suggests that the valorization of plant-based oil industry waste and by-products for use in the food industry could substantially reduce malnutrition and poverty, generate favorable health outcomes, mitigate environmental concerns, and enhance economic profit in a sustainable way by developing health-promoting, environmentally sustainable food systems.

## 1. Introduction

The food, agriculture, and oil industries are among the largest producers of organic waste globally [1]. Thus, food waste/by-products represent one of the most urgent global challenges, with far-reaching environmental, economic, and social consequences. It is estimated that one-third of all food produced for human consumption, roughly 1.3 billion tons annually, is lost or wasted [2,3]. Among the various food categories, the highest losses from post-harvest to distribution occur in roots, tubers, and oil-bearing crops (25%), followed by fruits and vegetables (22%), meat and animal products (12%), and cereals and pulses (9%) [4]. Moreover, around 20% of annual global food waste is attributed to oil-bearing crops [5].

**Figure 1:**
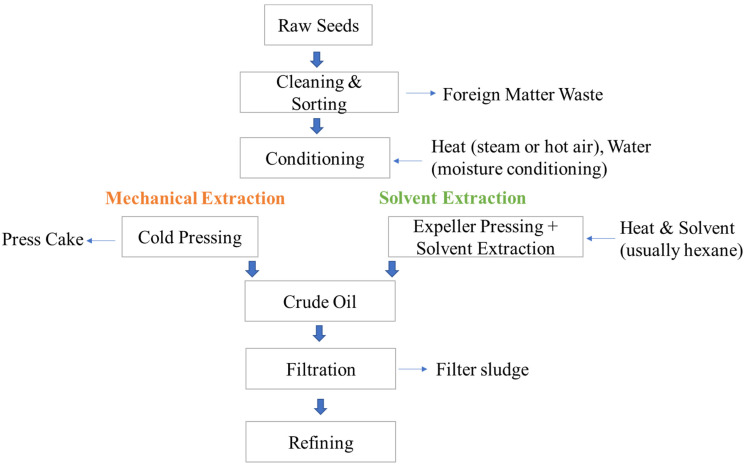
Generalized process flow diagram for edible oil production from plant-based sources.

The edible oil sector is one of the most significant branches of the global food industry, utilizing a wide variety of plant sources processed via various extraction techniques. According to FAOSTAT data, global edible oil production exceeded 200 million tons in 2022. Palm and palm kernel oil account for 41.1%, followed by soybean oil (27.4%), rapeseed/canola oil (12.6%), and sunflower oil (9.6%) [6]. While most plant-based oils are derived from seeds or fruits using mechanical pressing, solvent extraction, or a combination thereof, oils such as palm and olive are obtained by pressing the soft fruit pulp (Figure 1) [7]. Despite varying oil yields based on crop type and processing, the oil content of most raw materials remains moderate—for instance, soybean (16–22%), rapeseed/canola (40–48%), sunflower seed (42–62%), and palm fruit (30–40%) [8]. Consequently, a substantial proportion of the biomass—often more than half—remains as residual press cake or meal after extraction. These residues, commonly used as animal feed [8] or disposed of via composting, landfill, or incineration [2], are increasingly being explored for their potential in biofuel, bioadsorbent, biopolymer, and bioplastic production [9,10,11].

**Figure 2:**
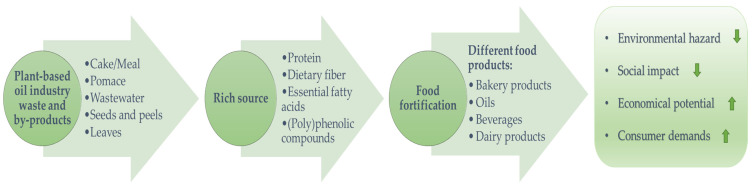
The potential positive effects of the sustainable utilization of plant-based oil waste/by-products as natural bioactive compounds in food applications.

Beyond their current applications, the generated waste/by-products of oil production offer substantial untapped potential due to their rich composition of proteins, fibers, and bioactive compounds. In 2021, the Food and Agriculture Organization (FAO) reported that 702–828 million people were suffering from hunger worldwide [12]. This number is also expected to rise in tandem with global population growth, projected to surpass 9 billion by 2050 [13]. Against this backdrop, the valorization of oil production waste as a low-cost, nutrient-rich raw material could significantly contribute to addressing food insecurity and malnutrition. These by-products are increasingly being recognized as valuable economic resources for the development of functional foods with enhanced nutritional and health-promoting properties [14]. The recovery and utilization of these materials align with the principles of a sustainable circular bioeconomy and the “zero waste model” promoted by the Agenda 2030 framework [15]. This approach not only supports environmental and economic sustainability but also responds to growing consumer demand for healthier and more sustainable food systems (Figure 2). At this point, the extraction of bioactive compounds from food waste/by-products represents a significant opportunity to fortify food products with natural additives, thereby promoting human health in a sustainable manner. However, among the extraction techniques, conventional methods have several drawbacks, including higher toxicity, environmental pollution, high energy consumption, and limited compatibility with the clean-label movement. Thus, there is growing interest in innovative green extraction techniques that are both compatible with green chemistry principles and yield-effective for recovering bioactive compounds from various natural sources [16,17]. Innovative extraction and processing techniques (such as ultrasound, microwave-assisted methods, enzymatic hydrolysis, natural deep eutectic solvents (NADES) extraction, etc.) have several advantages. Reducing solvent consumption and extraction time, improving extraction efficiency, and enabling the use of greener solvents are among the main advantages, leading to more effective, cleaner, and greener modern methods [2,18]. The growing awareness of the advantages of green extraction and processing techniques is leading to the growing potential of extracted bioactive compound usage in the production of functional foods, nutraceuticals, and sustainable materials.

Over the past decade, considerable research has explored the recovery of bioactive compounds from agro-industrial waste streams. Numerous reviews have addressed the valorization of general plant-based by-products (e.g., fruit peels, vegetable residues, and cereal bran) [19,20]. However, by-products generated from the mechanical or solvent extraction of oil-rich plant materials in the vegetable oil industry have received comparatively less attention in integrated and cross-comparative reviews. While some studies have examined individual sources such as olive pomace [21,22] or rapeseed meal [23], there is a lack of comprehensive evaluations that systematically assess the extraction, composition, bioaccessibility, and food applications of bioactives recovered from oil industry by-products across multiple oil types. These include the processing residues of olive, palm, avocado, coconut, canola/rapeseed, sunflower, flaxseed, and sesame seed oils, which are rich in phenolics, tocopherols, lignans, and bioactive peptides, yet vary considerably in their composition and functional potential. The existing literature often focuses on a single oil source, compound class (e.g., phenolics or proteins), or technological step (e.g., extraction or formulation), without linking these aspects to digestion stability, encapsulation strategies, and food integration.

The present review specifically focuses on the waste and by-products generated during the industrial processing of oils of plant-based origin and aims to explore the promising potential of them as alternative sources of bioactive and nutritious compounds. It highlights recent examples of innovative techniques for the extraction of natural bioactive compounds from underutilized plant-based oil processing waste/by-products and food fortification using such materials over the past decade and critically discusses the challenges, limitations, and future perspectives related to their incorporation into food systems.

## 2. Valorization of Waste/By-Products from Fruit-Based Oil Processing

**Figure 3:**
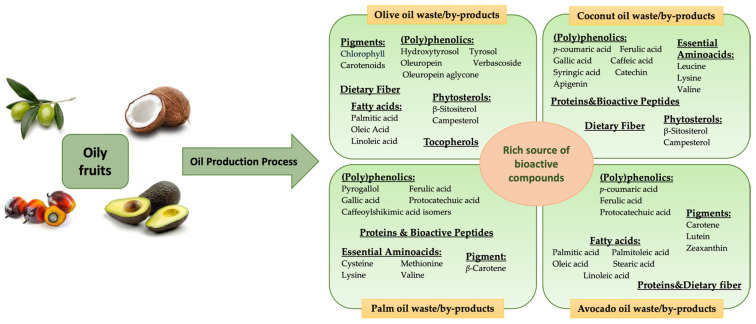
The bioactive potential of fruit-based oil processing waste/by-products.

Fruit-based oils (olive, avocado, coconut, and palm oil) are widely utilized in the food, cosmetic, and pharmaceutical industries due to their rich composition of bioactive compounds. However, their production generates substantial amounts of by-products, which are often underutilized or discarded, contributing to environmental burdens. Recent research has highlighted the potential of these residues as valuable sources of functional ingredients with antioxidant, antimicrobial, and health-promoting properties (Figure 3). Through green extraction technologies and innovative processing methods, these by-products can be transformed into high-value components for use in functional foods, nutraceuticals, and sustainable bioproducts, supporting a circular economy in the fruit oil sector.

### 2.1. Olive Oil Processing Waste/By-Products

The olive tree (*Olea europaea* L.) is a slow-growing evergreen native to the Mediterranean region, capable of living up to 1000 years. To fully appreciate its global significance, it is important to consider both its traditional cultivation areas and recent expansions into new regions. Traditionally cultivated in countries such as Spain, Greece, Italy, and Portugal, olive tree cultivation has recently expanded to regions like the United States, Argentina, New Zealand, and Australia [13,21,22,24]. Reflecting this expansion, global olive production in 2022 reached approximately 21.4 million tonnes, resulting in 2.74 million tonnes of olive oil. This corresponds to an average oil yield of 12.8% from raw olives. Spain, Italy, and Greece remain the dominant producers of olive oil [6]. However, between 2005 and 2020, non-EU Mediterranean countries (such as Tunisia, Turkey, and Morocco) also experienced significant growth in olive oil production [24,25,26].

Olive oil, a cornerstone of the Mediterranean diet, has been consumed since ancient times due to its ease of extraction and favorable nutritional profile [27]. The health benefits associated with olive oil have driven its increasing global demand. The health benefits of olive oil consumption are largely credited to its rich composition of mono-unsaturatedfatty acids and bioactive compounds with antioxidant, anti-inflammatory, antibacterial, and anticancer properties [25,28,29]. Reflecting this surge in demand, the global olive oil market has experienced remarkable growth over the past six decades. With a threefold expansion, the production of olive oil reached a volume of approximately 3.1 million tonnes and a market value of USD 14 billion in the 2021/22 season [22].

**Figure 4:**
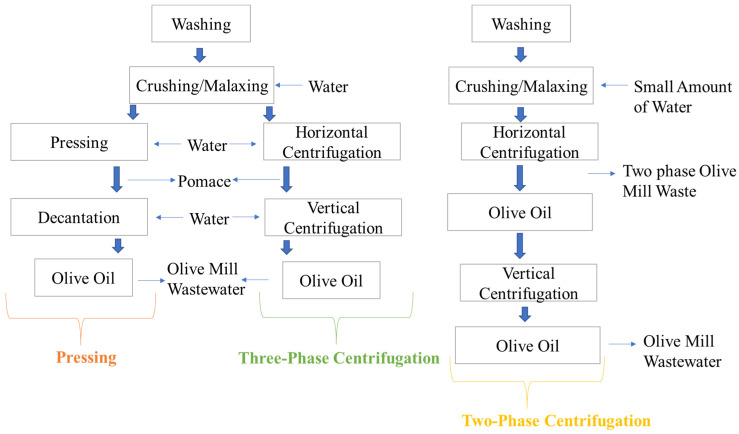
Flow diagram of olive oil production.

Fresh olives contain approximately 17–30% oil by weight, mostly stored in the mesocarp and epicarp, with only 1% in the kernel [27]. Understanding the extraction process provides insight into the quality and classification of olive oil. Olive oil is extracted through a series of steps including fruit cleaning, paste preparation, and oil separation from solid (pomace) and liquid phases via gravity or centrifugation (Figure 4) [27,30]. Common extraction systems include the traditional discontinuous press. In addition, two-phase and three-phase centrifugal processes are also widely used [31,32,33]. Oil classification depends on oxidation parameters such as fatty acid composition, peroxide value, and specific extinction coefficients (K_232_ and K_270_ values, corresponding to conjugated dienes and trienes, respectively) related to the production process. Olive oil is categorized as follows: extra virgin olive oil, virgin olive oil, ordinary virgin olive oil, lampante virgin olive oil, or refined virgin olive oil [24].

During production, substantial volumes of waste and by-products are generated—including olive pomace (skins, pulp, and pits), olive mill wastewater, and pruning residues (leaves and branches) [13,33]. The environmental impact of these by-products has sparked interest in their potential valorization. The three-phase system uses up to 50 L of water per 100 kg of olive paste, resulting in an estimated global generation of 10–30 million m^3^ of wastewater annually [32,34]. Furthermore, 35–40 kg of olive pomace is produced per 100 kg of olives processed. These by-products must be managed swiftly due to their environmental impact. However, they contain valuable compounds (such as phenolics, pectins, insoluble dietary fiber, proteins, and sugars) [35]. For example, olive pomace flour has been reported to contain 12.0% protein, 15.8% fat, 58.0% total dietary fiber, and 14.04 mg gallic acid equivalent (GAE)/g dw of phenolics [36]. Olive stone powder has been found to contain 5.1% protein, 7.6% fat, 53.2% fiber, 80.0% carbohydrates, and 0.37 mg GAE/100 g dw phenolics [37]. Such findings underscore the potential for valorizing olive oil waste/by-products across the food, pharmaceutical, and cosmetic industries.

Among the bioactives retained in these by-products, phenolic compounds are of particular interest. Only 1–2% of the olive fruit’s phenolics are transferred into the oil, with the majority remaining in the pomace, wastewater, and leaves. These by-products contain valuable compounds such as polyphenols, pigments (e.g., chlorophylls and carotenoids), tocopherols, fatty acids, phytosterols, and squalene. Many of these are useful in the food and pharmaceutical industries [27,33,38]. For instance, in terms of polyphenolic compounds, the most prevalent include hydroxytyrosol, tyrosol, oleuropein, oleuropein aglycone, and verbascoside in olive pomace [25]. Moreover, Ronca et al. [39] compared twelve Portuguese olive leaf samples. The potential of antioxidant activity of bioactive compounds in olive leaf was confirmed with total antioxidant activity assays. In addition, ORAC results highlighted that antioxidant response also depends on compound reactivity due to higher levels of slow-reacting antioxidants (like quercetin and rutin). Overall, CUPRAC proved effective in reflecting the polyphenolic contribution to antioxidant potential.

Recently, research has emphasized green extraction techniques. For instance, Antónia Nunes et al. [40] compared multi-frequency multimode modulated ultrasonic extraction to conventional solid–liquid extraction from olive pomace. The ultrasonic method yielded significantly higher antioxidant recovery (up to 402 μg GAE/mL) and hydroxytyrosol levels (283.4 mg/100 g dw), surpassing traditional methods. Similarly, pressurized liquid extraction achieved a higher total phenolic content (1659 mg GAE/kg dw) compared to solid–liquid extraction (281.7 mg GAE/kg dw). The major phenolics in pressurized liquid extracts included phenolic alcohols (42.5%), secoiridoids (26.4%), and flavonoids (16.4%). The high levels of phenolic alcohols were attributed to the thermal degradation of secoiridoids into simpler compounds, followed by several reactions (such as oxidation, hydration, and loss of the carboxylic and carboxymethyl groups) during pressurized liquid extraction [41]. Studies on olive mill wastewater have shown that maceration in methanol yields higher contents of total polyphenols (22.97 g GAE/100 g dw), flavonoids (2.34 g quercetin equivalent: QE/100 g dw), and tannins (2.47 g catechin equivalent: CE/100 g dw) than liquid–liquid extraction. Quinic acid was identified as the most abundant phenolic compound in both extraction methods [42].

Natural deep eutectic solvents (NADES) have also gained attention. Extraction temperature and solid–liquid ratio significantly influenced the extraction of polyphenolics from the olive pomace, while water content improved antioxidant activity up to an optimal level. A NADES mixture of lactic acid–glucose (5:1) with 68% water content at 80 °C showed the highest phenolic content (15.56 mg GAE/g dw) and antioxidant activity in olive pomace extraction. It outperformed both water- and ethanol-based extraction systems [43]. Chanioti et al. [44] also evaluated various innovative extraction methods using various solvent systems, including ethanol, methanol, and NADES. The most effective method for extracting phenolic compounds from olive pomace was determined to be the use of NADES solvents (particularly choline chloride–caffeic acid and choline chloride–lactic acid mixtures) in homogenization-assisted extraction at 60 °C/12,000 rpm and ultrasound-assisted extraction at 60 °C. Among the tested NADES formulations for olive leaf phenolic extraction, the lactic acid–glucose-based solvent exhibited the highest extraction efficiency for total phenolics, particularly enhancing the recovery of tyrosol derivatives and hydroxytyrosol. In contrast, choline chloride–glycerol mixtures favored the extraction of flavonoid glycosides (such as luteolin-7-O-glucoside and apigenin derivatives), while choline chloride–urea showed moderate performance, with better selectivity towards phenolic acids (like caffeic and coumaric acids) [45]. Furthermore, a better correlation was reported between phenolic content and antioxidant activity (based on DPPH, FRAP, ABTS, and CUPRAC assays) when using choline chloride and citric acid as a green solvent compared to conventional solvents [46].

Alternative green solvents like hydrogen-rich water and magnesium-enriched water have also shown promise. Hydrogen-rich water yielded the highest total phenolic (49.2 mg GAE/g), flavonoid (46.3 mg QE/g), and antioxidant activity (DPPH: up to 22.6 mg ascorbic acid equivalent/g), with a 34.4% extraction yield. It notably increased the content of several key phenolics (such as trans-ferulic acid and rosmarinic acid) and uniquely enabled catechin detection [47]. It can be concluded that the use of natural and green solvents has great potential for the valorization of bioactive compounds from waste and by-products in a variety of industries.

Olive oil waste and by-products are of significant interest due to their fatty acid profile and vitamin E content. They are characterized by a high proportion of mono-unsaturated fatty acids—especially oleic acid—and polyunsaturated fatty acids. Antónia Nunes et al. [40] analyzed the lipid and vitamin E composition of olive pomace. Oleic acid was identified as the predominant fatty acid at 75%, followed by palmitic (10%), linoleic (9%), and stearic acids (3%). The major vitamin E form was detected as *α*-tocopherol (7.64 mg/100 g dry weight (dw)), while *α*-tocotrienol, *β*-tocopherol, and ***γ***-tocopherol were present in smaller amounts. These findings were corroborated by Pasten et al. [48], who also studied the impact of drying temperatures on olive pomace composition. Drying at 60 °C notably increased linolenic acid by up to 12.7-fold compared to fresh samples, whereas palmitic and oleic acids decreased when drying exceeded 60–70 °C. Vitamin E levels remained stable throughout the drying process.

Olive solid waste predominantly consists of lignocellulosic compounds, with variable levels of lignin (30.0–41.6%) and polysaccharides (35–49%, including cellulose, hemicellulose, pectic polymers, etc.) [27]. A detailed compositional analysis [49] reported lignin as the most abundant structural carbohydrate (43.95 g/100 g dw), followed by hemicellulose (11.29 g/100 g), cellulose (9.55 g/100 g), and xylose (8.03 g/100 g). Additionally, crude olive pomace contained 46.48 g/100 g neutral detergent fiber, 31.06 g/100 g acid detergent fiber, and 3.23 g galacturonic acid equivalents/100 g total soluble pectin. Consequently, dietary fiber derived from olive oil waste is a valuable functional ingredient. Rubio-Senent et al. [50] extracted pectin from olive pomace via steam treatment at 160 °C for 30, 45, and 60 min. The purified extracts showed low water holding capacity (0.34–1.87 g water/g sample), high oil holding capacity (2.99–6.17 g oil/g sample), moderate emulsifying activity (50.33–54.19%), and emulsion stability (14.58–19.09%) comparable to commercial apple pectins. In vitro assays also revealed significant bile–acid binding and a glucose retardation index similar to that of citrus pectins.

Micronization effects on olive pomace fractions, separated by particle size (>2 mm: F1, <2 mm: F2), were evaluated to assess changes in dietary fiber composition and functional properties [51]. The finer F2 fraction had a higher amount of soluble dietary fiber. It also showed greater water and oil holding capacities, solubility, and cation exchange capacity compared to F1 fraction. Additionally, micronization resulted in increased soluble dietary fiber, reduced lignin content, and enhanced cellulose and hemicellulose levels, improving overall functionality. Conversely, Aslan Türker and Işçimen [52] reported that reducing the particle size of insoluble dietary fiber from olive pomace decreased water and oil holding capacities. The same study also evaluated the stabilization of Pickering emulsions using insoluble dietary fiber from olive pomace. The olive pomace fiber provided protection against agglomeration and good stability, regardless of particle size.

Olive oil wastes also represent an emerging source of plant-based proteins, although their protein content is generally low (1–4% in olive pomace [53]; ~5% in olive stone powder) [37]. Olive leaves are more extensively studied for protein extraction. An enzyme-assisted method using 30% acetonitrile and 5% Celluclast^®^ (at pH 5.0 and 55 °C for 15 min) yielded total protein content between 1.87 and 6.64 mg/g across different olive leaf varieties [54]. Electrophoretic analysis identified a 63 kDa protein band corresponding to oleuropein *β*-glucosidase in Solà, Hojiblanca, and Picual cultivars.

Comparing extraction methods, ultrasound-alkaline-assisted extraction outperformed conventional alkaline and industrial heat-coagulation techniques for olive leaf protein isolation [13]. This method achieved an extraction yield of 11.67% with 51.2% protein content, and the highest essential amino acid concentration (29.10 mg/100 mg protein). The most abundant essential amino acids were isoleucine (4.02–4.58 mg/100 mg), leucine (4.71–6.22 mg/100 mg), valine (3.77–4.91 mg/100 mg), and phenylalanine + tyrosine (5.81–6.77 mg/100 mg). Additionally, ultrasound-assisted extraction improved protein solubility (92%), foaming capacity (72.5%), foaming stability (84.5%), in vitro digestibility (62.2%), and thermal stability. However, another study found that alcohol washing, heat treatment, and de-oiling reduced olive leaf protein content from 80% to 40%, altering protein conformation and functionality [55]. These results underscore the potential of olive leaves as a sustainable protein source if extraction methods are optimized.

As olive oil and Mediterranean diet foods gain popularity, nutrient- and bioactive-rich olive oil wastes and by-products are being increasingly used in fortified functional foods. The effects of these incorporations are extensively studied, highlighting the potential of olive by-products for innovative food applications.

Bakery products are among the most extensively studied food types due to their simple and flexible formulation and widespread consumption. For instance, Simsek and Süfer [36] incorporated olive pomace flour at varying levels (5%, 7.5%, and 10%) into breadstick formulations made with wheat and whole-wheat flour. Fortification with olive pomace flour significantly increased total phenolic content and antioxidant capacity. The highest phenolic content was observed at the 10% addition level for both wheat and whole-wheat breadsticks. Sensory attributes remained satisfactory up to 10% fortification, and the formulation containing 7.5% olive pomace flour was identified as optimal [36]. Similarly, Jahanbakhshi and Ansari [37] investigated the effect of adding olive stone powder (at 15%, 25%, and 35% levels) to sponge cake. This supplementation notably enhanced nutritional properties, with antioxidant phenolic compounds increasing from 89.14 to 739.23 μg/mL and fiber content rising from 0.67% to 8.60%. The physicochemical and textural characteristics of the sponge cake were also affected by the inclusion and concentration of olive stone powder. Sensory evaluation indicated no significant differences in taste, aftertaste, color, or odor between fortified cakes and the control. The fortified cake containing 15% olive stone powder was identified as most closely resembling the control sample. Furthermore, Simonato et al. [56] evaluated the substitution of durum wheat semolina with olive pomace at 5% and 10% levels in pasta production, assessing its effects on technological and nutritional properties. The addition of olive pomace increased cooking loss, water absorption, firmness, and adhesiveness, while reducing optimal cooking time. Notably, olive pomace decreased rapidly digestible starch and increased slowly digestible and resistant starch fractions, without significantly altering the predicted in vitro glycemic index. The fortification also led to considerable increases in total phenolic compounds and dietary fiber content [56]. These findings collectively highlight olive oil by-products as promising ingredients for developing bakery products with enhanced phenolic content and dietary fiber, offering both health benefits and viable technological alternatives.

Olive oil wastes and by-products are also utilized as natural preservatives to improve the quality and extend the shelf life of highly perishable, lipid-rich foods. Panza et al. [57] demonstrated the valorization of dried olive paste in breading ready-to-cook cod sticks. Enrichment with olive paste elevated total phenolics, flavonoids, and antioxidant activity in the breaded fish samples. Microbial analysis confirmed the antimicrobial effect of phenolic compounds from olive paste. In parallel, the fortification resulted in extending the product shelf life by three days (up to 12 days) without compromising sensory properties. In another study, Karadag et al. [58] explored the production of phenolic-enriched lecithin via cloud point extraction from olive mill wastewater for direct food application. This enriched lecithin was used to fortify a vegan salad dressing, significantly enhancing its oxidative stability compared to formulations using non-enriched lecithin. Similarly, enriching olive oil with a micellar dispersion of phenolics extracted from olive mill wastewater via lecithin-based cloud point extraction improved polyphenol content (by up to 42.2% at a 0.5% addition level) and extended shelf life without altering organoleptic qualities [59]. Additional examples of food fortification using olive oil processing wastes and by-products are summarized in Table 1.

### 2.2. Palm Oil Processing Waste/By-Products

Oil palm (*Elaeis guineensis*) is a major agricultural crop in Malaysia and Indonesia. Together, these countries produced about 84% of global crude palm oil in 2023/2024, totaling approximately 78 million tons [67]. Two primary oils are extracted from the palm fruitlets, which cluster on the fresh fruits: crude palm oil from the mesocarp and palm kernel oil from the endosperm of the seeds [68]. Palm oil ranks among the most valuable edible oils globally, representing nearly 60% of worldwide oilseed exports. The global palm oil market is projected to reach USD 25.3 billion by 2025 [69]. Despite its economic significance, only about 10% of the palm tree biomass is used for oil extraction. The remaining 90% consists of vegetative material [68], producing large amounts of waste (such as palm kernel cake, pressed fiber, empty fruit bunches, mill effluent, kernel shells, trunks, and leaves) [70,71,72].

These waste/by-products are valuable due to their rich macro- and micronutrient content, making them important sources of nutrition and energy. For instance, Chang et al. [73] reported that defatted palm kernel meal contains approximately 7.87% lipid, 3.76% ash, 54.8% protein, 21.5% total carbohydrate, 8.61% moisture, and 3.46% crude fiber. The phenolic content was measured at 28.9 mg GAE per 100 g [73]. As a result, palm oil waste is being increasingly used in biorefineries to create high-value products. This approach supports both economic and environmental sustainability. Beyond biorefining, these wastes have considerable potential for conserving food resources and developing functional foods due to their nutritional composition.

Palm oil waste and by-products contain substantial amounts of bioactive compounds (such as phenols, sterols, proteins, and glycolipids), which exhibit anticancer, anti-inflammatory, and antioxidant activities [71]. Accordingly, numerous studies have focused on the extraction, purification, and bioactivity assessment of these compounds to explore the valorization potential of palm oil processing residues.

A significant by-product is palm kernel cake, widely used as feed for cattle and poultry. Palm kernel cake contains vitamins, minerals, and approximately 20% crude protein [74]. Proteins in palm kernel cake can be effectively extracted using alkaline solubilization or enzyme-assisted methods. Enzymatic hydrolysis by proteases (such as papain, alcalase, and trypsin) produces bioactive peptides with potent antibacterial, antihypertensive, and antioxidant properties [74]. Important palm-kernel-cake-derived peptides (e.g., AWFS, WAF, YLLLK, YGIKVGYAIP, and LPWRPATNVF) demonstrate significant bioactivity at low concentrations [74,75]. For example, Chang et al. [73] reported that defatted palm kernel meal contained 54.8% protein, while protein isolates reached 75.6%. The isolates and hydrolysates had improved amino acid profiles, especially higher levels of cysteine, methionine, valine, and lysine, compared to soluble fractions. Another study showed that alcalase-derived protein hydrolysates from palm kernel meal had strong radical scavenging activity. After 60 min of hydrolysis, the IC_50_ values were 5.73 μg/mL for DPPH and 7.84 μg/mL for ABTS assays. These hydrolysates were non-toxic to mouse L929 fibroblast cells and protected them against H_2_O_2_-induced oxidative damage [76]. Similarly, Zarei et al. [75] found that all enzymatically derived palm kernel cake hydrolysates showed effective angiotensin-converting enzyme inhibition (22.9–70.9%), with papain hydrolysate exhibiting the strongest effect (70.9%). When administered at 75 mg/kg body weight to hypertensive rats, the papain hydrolysate significantly stabilized blood pressure. These findings underline palm kernel cake as a valuable protein and bioactive peptide source with important physiological and functional roles in food enhancement.

Carbohydrates constitute the majority of palm oil biomass, with lignocellulose—a composite of cellulose, hemicellulose, and lignin—being a major component [69,77]. The lignocellulosic fraction offers great potential for biorefinery applications such as bioenergy, bio-absorbents, biopolymers, and bio-sugars. Research on the extraction and modification of palm biomass lignocellulose also holds promise for the food and pharmaceutical industries. For example, soluble polysaccharides extracted from defatted palm kernel cake using water, citric acid, or NaOH were characterized and evaluated for prebiotic potential [78]. These polysaccharides comprised mannose, galactose, glucose, arabinose, xylose, and rhamnose, with total carbohydrate contents of 57.11%, 56.94%, and 50.95%, respectively. They exhibited high resistance to hydrolysis (>96%) in simulated human gastric juice and promoted the proliferation of probiotic strains *Lactobacillus plantarum* ATCC 8014 and *Lactobacillus rhamnosus* ATCC 53103. Similarly, oligosaccharides isolated from palm kernel cake using a neutral detergent showed prebiotic effects. These effects were mainly attributed to low-molecular-weight mannobiose and mannotriose [79].

Additionally, palm fruit fibers extracted by supercritical fluid extraction post-oil-removal contained 59.26% total dietary fiber (56.24% insoluble and 0.49% soluble) and exhibited notable antioxidant activity. This fiber fraction was comparable to dietary fibers used in existing food products, suggesting potential applications as a functional food ingredient [80]. Further exploration of palm oil biomass as a dietary fiber source could foster the development of delivery systems, wall materials, food packaging, and novel bio-based food formulations.

Other significant biologically active substances present in substantial amounts in palm oil biomass include phytochemicals (such as phenolic compounds, carotenoids, sterols/polysterols, quinones, and others), making palm oil biomass an excellent source of these compounds [71]. One study examined the phenolic content of various palm oil biomass fractions. Palm kernel cake had the highest total phenolic level (5.19 mg GAE/g of dw), while empty fruit bunches had the lowest (1.79 mg GAE/per g dw) [81]. The same study assessed the effects of extraction time and liquid-to-solid ratio on phenolic extraction, finding that a 40:1 solvent-to-solid ratio for 20 min yielded the highest phenolic content in palm kernel cake (5.35 mg/g). The phenolics identified in palm kernel cake extracts included pyrogallol, 4-hydroxybenzoic acid, gallic acid, and ferulic acid [81]. Another investigation reported that phenolic compounds extracted from palm oil mill effluent contain high concentrations of caffeoylshikimic acid isomers (10,800 mg/kg total), *p*-hydroxybenzoic acid (7000 mg/kg), and protocatechuic acid (6000 mg/kg). These phenolics demonstrated significant protective bioactivity against cardiovascular disease in preclinical models without toxicity or teratogenic effects [82]. Additionally, phenolics extracted from palm kernel cake were fractionated into cell-wall-bound and free methanol-soluble forms. It was revealed that 29.56% (210.2 μg/g dw) was cell-wall-bound, while 70.44% (500.9 μg/g dw) was freely soluble in methanol [83].

Green extraction techniques and environmentally friendly solvents have been increasingly applied to recover bioactives from oil palm waste. Nomanbhay et al. [84] demonstrated that microwave-assisted extraction enhanced phenolic recovery from palm oil mill effluent, increasing yield by 15.8% and reducing extraction time by 98.75% compared to conventional maceration. Combining microwave-assisted extraction with enzymatic hydrolysis further improved phenolic yield to 221.9 mg GAE per 10 g of lyophilized palm oil mill effluent, compared to 190.9 mg GAE without enzymatic treatment.

Ultrasound-assisted extraction from palm pressed fiber identified several bioactives, including *β*-sitosterol (2.77–8.09 mg/g), *α*-tocopherol (2.92–9.46 mg/g), squalene (1.42–1.73 mg/g), total phenolics (0.85–1.07 mg GAE/g), and carotene (2.53–4.07 mg/g). All showed antioxidant activity against synthetic (DPPH, ABTS) and biological (OH) radicals [85]. In a recent innovative study, Ling Tang et al. [86] used palm oil mill effluent as a novel extraction solvent combined with 5% (*w*/*w*) NaOH as an alkali catalyst to extract lignin and phenolic compounds from oil palm empty fruit bunch fiber. This method yielded the highest lignin content (24.2% ± 0.36%) and phenolic concentration (2.4 g/L). Fatty acid profiling revealed that lauric, palmitic, and oleic acids accounted for 80% of the total fatty acids extracted from palm pressed fiber using supercritical CO_2_ and compressed liquefied petroleum gas solvents [87]. Similarly, palmitic, oleic, linoleic, and stearic acids were the predominant fatty acids found in palm oil mill effluent [88]. Palm pressed fibers were confirmed to contain antioxidants such as *α*-tocopherol, squalene, *β*-carotene, and *β*-sitosterol [87], while palm oil mill effluent was rich in pro-vitamin A, vitamin E, squalene, and phytosterols, contributing to high total flavonoid content and moderate antioxidant activity [88]. Collectively, these findings highlight the potential of palm oil biomass for developing novel functional foods that enhance nutritional quality, reduce environmental impact, and generate economic value in the food industry.

The incorporation of biologically active compounds derived from oil palm waste into various food products has the potential to enhance their nutritional value and texture significantly. To explore the valorization potential of palm oil waste in food preparation, hydroxypropyl methylcellulose (HPMC) extracted from palm oil empty fruit bunches was evaluated as a coating material for French fries at concentrations of 0%, 1%, 2%, and 3%. The application of a 3% HPMC coating reduced oil absorption by 16.1%. However, increasing the HPMC concentration reduced the fat content due to stronger bonds forming between the coating polymer and the surface of the fries. This was accompanied by an increase in moisture content and a softer texture [89].

Microencapsulation of ginger (*Zingiber cassumunar* Roxb) essential oil using oil palm trunk waste fibers was also investigated [90]. However, the fibers alone caused a reduction in terpinen-4-ol content, indicating that encapsulation with the trunk fiber matrix alone was insufficient. Combining these fibers with chitosan was suggested as a more effective encapsulation strategy. Conversely, modified cellulose derived from palm oil empty fruit bunches demonstrated strong potential in stabilizing Pickering emulsions against coalescence. Water retention tests and emulsion stabilization experiments confirmed that this modified cellulose could be valuable for applications in packaging, food, drug delivery, and pharmaceuticals [91].

In an antioxidant fortification study, sunflower oil was supplemented with 0.8% palm kernel cake extract and 0.46% palm pressed fiber extract. Butylated hydroxyanisole (BHA) and an untreated control were used as references [81]. The palm pressed fiber extract increased the oil’s induction time by up to 30%, while the palm kernel cake extract achieved nearly a 60% increase. These results are comparable to those obtained with BHA, highlighting the potential of palm kernel cake extract despite containing substances that may act as pro-oxidants.

Compared to other oil processing by-products, research on the valorization of palm oil biomass for food fortification and functional food development remains limited. Nonetheless, palm oil waste and by-products present promising opportunities for the food industry and researchers aiming to enhance nutritional quality, sustainability, and economic value. Further investigation into the design of new food formulations and the bioaccessibility/bioavailability of bioactive compounds in palm oil waste and by-products within complex food matrices will provide more information and opportunities for utilizing underutilized palm oil production biomass in the food industry and science.

### 2.3. Avocado Oil Processing Waste/By-Products

Avocado (*Persea americana* Mill), a tropical and subtropical fruit, is native to Mexico and Central America. Mexico is the world’s leading producer, accounting for over 28% of global production. Other major producers include Colombia, Peru, the Dominican Republic, Kenya, Indonesia, and Brazil [92,93,94,95]. In recent years, avocado consumption has risen sharply. This is largely due to its status as superfood, driven by its excellent nutritional profile, rich phytochemicals, and proven health benefits. Correspondingly, global production rose by 4.8%, from 8,570,284 tons in 2021 to 8,978,275 tons in 2022 [94]. While fresh fruit remains the primary form of consumption, industrial products derived from avocado, particularly guacamole and avocado oil, have seen significant growth [92,96]. Although avocado oil production in Colombia remains limited—since most avocados are sold fresh or processed into guacamole—the avocado oil market was valued at approximately USD 484.6 million in 2020 [97].

The high oil content of avocado is primarily located in the mesocarp (about 60%), followed by the outer skin and seed. Consequently, avocado oil is mainly extracted from the fruit’s flesh via mechanical (cold pressing) and chemical (organic solvent) methods. During oil extraction, large amounts of waste are produced. This includes seeds, peels, pomace, and wastewater. These by-products contain invaluable compounds such as phytochemicals, fiber, and other bioactives. Depending on the avocado variety, they can make up about 25% of the fresh fruit weight [92,98]. Proximate analysis of cold-pressed avocado oil by-products revealed that the skin contains a high dietary fiber content (81.4% dw), while the seeds, pomace, and wastewater are rich in carbohydrates, protein, and lipids, respectively [99]. However, these compositions may vary based on avocado variety and processing methods. Moreover, these by-products are a valuable source of bioactive phytochemicals (such as phenolic compounds, carotenoids, sterols, and tocopherols) [92,100]. Thus, the waste/by-products of avocado oil production show promise as a raw material and an eco-friendly source of valuable compounds for use in a variety of industries.

Avocado oil waste and by-products are recognized for their phytochemical richness, contributing to notable health-promoting properties and chemical diversity. This suggests their potential in the development of novel products with broad industrial applications. Therefore, the identification and extraction of key phytochemicals from avocado oil by-products have garnered significant scientific and industrial interest. For instance, Permal et al. [99] assessed the antioxidant potential of avocado oil by-products (seed, pomace, skin, and spray-dried wastewater) using three complementary assays at different pH levels. Total phenolic content in freeze-dried skin, seed, pomace, and wastewater from cold-pressed avocado oil extraction was measured as 13.7, 8.1, 3.6, and 1.6 g GAE/100 g powder, respectively. The CUPRAC method, which measures the reduction of Cu(II) to Cu(I), revealed notably high antioxidant activity in spray-dried powders, especially at higher inlet temperatures, indicating a temperature-dependent enhancement. While avocado skin showed the highest activity in FRAP and phosphomolybdenum assays, CUPRAC also highlighted strong reducing power in skin and seed extracts compared to flesh. These findings confirm the value of CUPRAC in capturing the polyphenol-related antioxidant activity of avocado by-products [99]. Similarly, Velderrain-Rodríguez et al. [101] reported that ethanolic extracts of avocado peels exhibited 2.4 times higher TPC and twice the antioxidant activity (measured by DPPH, ABTS, and FRAP assays) compared to seed extracts. Phenolic profiling revealed ferulic acid (392.45 mg/100 g) and *p*-coumaric acid (251.77 mg/100 g) as the predominant phenolics in peel extracts, whereas ferulic acid (555.32 mg/100 g) and catechin (247.47 mg/100 g) dominated seed extracts. Carotenoid analysis identified lutein (32.34 mg/100 g), *α*-carotene (3.61 mg/100 g), and *β*-carotene (6.84 mg/100 g) in peel extracts, while seed extracts contained only low lutein levels (0.16 mg/100 g). Both extracts contained *δ*-, *γ*-, and *α*-tocopherols, with peel extracts exhibiting a higher total tocopherol content (82.84 mg/100 g) than seeds (36.37 mg/100 g).

Zuñiga-Martínez et al. [102] investigated the bioactive composition, in vitro digestion, and in silico interactions of phenolics with cholesterol in avocado paste derived from defatted pulp, peel, and seed residues. Ferulic acid, *p*-coumaric acid, and protocatechuic acid were the major phenolics, contributing to a total phenolic content of 2.1 mg GAE/g dw. Most phenolics were covalently bound, requiring hydrolysis for release, with acid hydrolysis proving more effective than alkaline. *β*-Carotene (98.8 μg/100 g dw) and *α*-tocopherol (10,412 μg/100 g dw) were the most abundant carotenoid and tocopherol, respectively, with *γ*-tocopherol and zeaxanthin also present. Phenolic release and antioxidant capacity peaked during the gastric digestion phase. In silico analyses showed that ferulic acid had the strongest cholesterol interaction, suggesting a potential inhibitory mechanism on cholesterol intestinal absorption.

Extraction methods have been extensively studied for bioactive recovery. Microwave-assisted extraction was identified as an efficient green technique for polyphenol extraction from avocado peel [103]. Optimal conditions were 130 °C, 39 min, 36% ethanol, and a solvent-to-sample ratio of 44 mL/g, yielding 73.2 ± 3.8 mg GAE/g peel dw, nearly eight times higher than conventional solid–liquid extraction. Microwave-assisted extracts also demonstrated effective inhibition of matrix metalloproteinases (matrilysin, gelatinase A, collagenase-1) at low concentrations, comparable to potent inhibitors. Combining ultrasound with microwave extraction further enhanced phenolic recovery, achieving 166.3 ± 4.9 mg GAE/g dw and a yield of 25.3 ± 0.6%, while reducing energy and raw material use [104]. This combined method yielded higher total phenolic, flavonoid, and anthocyanin contents alongside superior antioxidant activity compared to ultrasound, microwave, or maceration alone. Rodríguez-Martínez et al. [105] evaluated deep eutectic solvents as eco-friendly alternatives to organic solvents for extracting phenolics from avocado peels. Choline chloride–lactic acid (1:3) and choline chloride–acetic acid–water (1:1:10) yielded the highest TPCs (~92 mg GAE/g dw), surpassing 96% ethanol extraction. Deep eutectic solvent extracts exhibited strong antibacterial activity against *Staphylococcus aureus*, *Streptococcus dysgalactiae*, *Escherichia coli*, and *Pseudomonas putida*. Della Posta et al. [106] also confirmed the efficacy of DES in the extraction of phenolic compounds from Hass avocado peel. The findings demonstrated that a higher extraction efficiency was obtained when a reduced amount of solvent and time was used at optimized conditions with DES (consisting of choline chloride and lactic acid) compared to the organic solvents traditionally used.

The lipid profile of avocado paste/cake from oil processing is dominated by monounsaturated fatty acids (MUFAs), followed by polyunsaturated (PUFAs) and saturated fatty acids. Oleic acid (C18:1, ω-9) is the most abundant, accompanied by linoleic acid (C18:2, ω-6), palmitic acid (C16:0), palmitoleic acid (C16:1, ω-7), *α*-linolenic acid (C18:3, ω-3), and stearic acid (C18:0) [102,107]. Neves et al. [108] characterized over 320 lipid molecular species across 21 classes in avocado peel and seed using liquid chromatography–tandem mass spectrometry, identifying four novel lipid classes. Drying methods (hot air, vacuum, and hot-air microwave) significantly reduced oleic acid content; however, hot-air drying preserved higher oleic and linoleic acid levels in pulp and peels, while hot-air microwave drying retained the highest *α*-linolenic acid content [109].

In addition to lipids, avocado oil by-products are also rich in carbohydrates, dietary fiber, and proteins. The dietary fiber content in avocado meal (from oil processing) was 10.4% soluble and 27.0% insoluble [107]. This study also demonstrated that avocado meal is a viable dietary fiber source for canine diets, comparable to traditional fiber sources like beet pulp and cellulose, without adverse health effects. To further explore fiber extraction, Barbosa-Martín et al. [110] compared two solvents for dietary fiber extraction from avocado seed residues, reporting extraction yields between 45.6% and 48.1% with similar fiber compositions. The extracted fiber fractions could also absorb up to four times their weight in water and six times in oil, indicating potential as functional food additives.

Martins et al. [3] isolated starch from avocado seeds with a 19.54% yield using 0.2% sodium metabisulfite solution. The starch exhibited oval granules with smooth surfaces, low protein and lipid content, and a high amylose content (39.56%). Functional properties (water activity, water absorption index, oil absorption capacity, water solubility, transmittance, and thermal stability) were detected to be suitable for processing. Thus, the extracted starch could be used in the processing of edible films and the manufacturing of biodegradable materials at temperatures of up to ~ 366 °C. In parallel with these findings, the extracted starch from avocado seeds was successfully used as a wall material for encapsulation of ginger essential oil [111].

Avocado oil by-products show promise as a protein source. However, research on their extraction and characterization is still limited. Wang et al. [112] investigated the physicochemical properties and functionality of an edible protein isolate derived from defatted avocado meal. The protein was found to be rich in essential amino acids, including threonine, valine, isoleucine, leucine, phenylalanine + tyrosine, lysine, and histidine. Compared to soy protein, avocado protein exhibited superior water and oil absorption capacities and stronger radical scavenging activity, albeit with lower in vitro digestibility. Furthermore, its application as an emulsifier significantly enhanced the stability of oil-in-water emulsions. These findings highlight the potential of avocado-derived protein from oil production waste to enhance the nutritional profile and functionality of food products. Nevertheless, further research is warranted to better understand the influence of extraction and processing conditions on the protein’s functionality, digestibility, and nutritional quality.

Growing consumer interest in avocado is fueled by its nutritional and health benefits. This trend offers a valuable opportunity to use avocado oil by-products in functional food development. These by-products offer a sustainable source of bioactive and nutrient-dense compounds, aligning with the goals of a circular economy in the food industry.

One critical consideration in formulating functional foods with food waste or by-products is consumer acceptance, alongside nutritional value and sensory quality. In this context, a study evaluated corn chips fortified with avocado paste—comprising defatted pulp, peel, and seed—at 2%, 6%, and 10% inclusion levels [113]. Fortification significantly enhanced the total phenolic content (from 0.93 to 3.56 mg GAE/g dw) and total flavonoids (from 1.17 to 6.61 mg QE/g dw), with corresponding improvements in antioxidant activity. Nutritionally, the fortified chips exhibited an improved lipid profile, increased dietary fiber, and elevated levels of key minerals and fatty acids (such as oleic, palmitic, and linoleic acids). Sensory evaluation revealed that chips fortified with 2% avocado paste were most preferred, receiving an 82% consumer acceptance rate. In another study by Campos-González et al. [114], avocado residual paste extract obtained via optimized microwave-assisted extraction was incorporated into artisanal pork ham at 0%, 0.5%, 1%, and 1.5% (*w*/*w*) levels [114]. Fortification enhanced the total soluble phenolic content by 13%, 24%, and 37% for the respective concentrations, with the formulation containing 1% extract receiving the highest sensory approval.

Avocado seeds have also been valorized in the production of ready-to-eat extruded snacks. Compared to extrudates made from brown rice and malted barley, avocado seed-based products showed significantly higher antioxidant activity and total phenolic content [115]. Considering safety aspects, the same study evaluated levels of potential toxins—amygdalin and persin—in fresh and extruded avocado seeds. The results indicated that amygdalin (2.6 × 10^−6^ mg/g) and persin (0.68 mg/g) in the final product were well below toxic thresholds, suggesting that these snacks are safe for consumption.

Moreover, avocado oil processing by-products have shown potential as natural food preservatives. Among avocado oil by-products, spray-dried avocado oil wastewater powders exhibited the highest antioxidant activity (between 8.8 and 12.6 g TE/100 g of powder) based on the CUPRAC assay, compared to the FRAP and phosphomolybdenum assays. Thus, spray-dried avocado wastewater powder added at 0.2% (*w*/*w*) to pork sausages demonstrated antioxidant effectiveness comparable to the synthetic additive sodium erythorbate (E316) at 0.04% (*w*/*w*), as measured by the thiobarbituric acid-reactive substances (TBARS) assay [99]. Similar antioxidant protection was observed when spray-dried avocado wastewater encapsulated with 5% whey protein concentrate was incorporated into pork fat cooked at 180 °C for 15 min [116]. These preservative effects are attributed to the presence of lipid-soluble antioxidants in the avocado waste matrix.

An overview of additional studies exploring the incorporation of avocado oil by-products into functional food formulations is provided in Table 2.

### 2.4. Coconut Oil Processing Waste/By-Products

Coconut (*Cocos nucifera* L.), a member of the Arecaceae family, is a major cultivated tree crop in tropical and subtropical regions. The leading coconut producers include Indonesia, the Philippines, and India, followed by Brazil and Sri Lanka [123,124]. The coconut industry has rapidly expanded in recent years, resulting in a diverse array of products (such as fresh coconut, coconut oil, copra meal, desiccated coconut, coconut water, virgin coconut oil, coconut milk, coconut sugar, and coconut cream) [125,126,127]. Among the various coconut products, coconut oil is one of the most popular and marketed coconut products.

Coconut oil is extensively consumed, especially in Sri Lanka and other parts of South Asia. Its high content of saturated fatty acids imparts remarkable thermal stability, making it ideal for high-temperature culinary applications such as frying and baking [128]. Although saturated fat raises health concerns, coconut oil is rich in medium-chain fatty acids—mainly lauric acid—which has unique physicochemical properties. It also contains bioactive compounds such as phenolics and sterols, contributing to various health benefits [128,129,130]. Reflecting its functional versatility, coconut oil accounted for approximately 50% of the global coconut product market in 2019 [127], and virgin coconut oil is expected to grow at a compound annual growth rate of 8.6% from 2023 to 2030 [131].

Coconut oil is mainly obtained using two methods: copra oil and virgin coconut oil production. Copra oil is extracted from dried kernels, then refined, bleached, and deodorized. For the production of virgin coconut oil, the shredded wet coconut kernel is exposed to pressure to extract the oil and coconut milk, which results in the formation of an emulsion. The emulsion is then subjected to various separation techniques to yield the desired product [123,132]. Different extraction methods, including low-pressure extraction, chilling, freezing and thawing, fermentation, centrifugation, enzymatic extraction, and supercritical fluid carbon dioxide, are preferred for various uses and applications in the production of coconut oil depending on the desired purity and yield of lauric acid [133].

Coconut oil production also generates large amounts of waste, such as press cake, pulp, wastewater, and both solid and gaseous residues [134,135]. In 2019 alone, the coconut oil industry produced approximately 1.9 million metric tons of pulp residue as a by-product [136]. Among the generated by-products, spent coconut meal or pellet—traditionally the primary by-product—stands out for its nutritional value, being rich in dietary fiber, protein, and essential nutrients. It is primarily processed into coconut flour, which offers potential health benefits [126,131]. From 2018 to 2022, the spent coconut flour market experienced steady growth, with a compound annual growth rate (CAGR) of 7.2%. By 2033, its market value is projected to reach USD 6325.6 million [131].

According to Fonseca-Bustos et al. [137], coconut flour derived from residues following virgin coconut oil extraction contains approximately 3.29% moisture, 3.75% ash, 22.71% protein, 44.42% crude fat, and 25.83% total carbohydrates. However, its composition may vary depending on the specific oil processing method and conditions. These findings underscore the potential for converting coconut oil by-products into functional food ingredients, offering both environmental and economic benefits through improved resource utilization [126].

As lignocellulosic residues, coconut industry by-products are often valorized within the biorefinery framework for diverse applications (e.g., civil construction, filtration, adsorption, ethanol production, and pyrolysis) [11]. Notably, they are also widely used in animal feed. Nonetheless, research increasingly highlights that these by-products—particularly spent coconut meal—represent a significant source of bioactive and nutritive compounds [126,131]. Their incorporation into food systems offers a sustainable strategy to enhance nutritional value while mitigating environmental impact.

Coconut oil cake is particularly notable for its protein content, averaging around 20%, making it a promising alternative protein source across various industries [138]. Nutritional profiling of edible coconut protein concentrate, obtained from wet processing residues, has revealed a high protein content (80.3%) and a complete amino acid profile. Among the essential amino acids, leucine, lysine, and valine were predominant, with glutamic acid being the most abundant overall [139]. Similarly, Rodsamran and Sothornvit [140] identified glutelin as the dominant protein fraction in both coconut oil and milk cakes. Their comparative study on the functional properties of protein powders from these sources showed that milk-cake-derived proteins exhibited superior water and oil absorption capacities, whereas oil cake proteins demonstrated enhanced foaming and emulsifying abilities. These findings suggest promising applications for coconut protein powders in food formulation. When developing food products with plant-based by-products as a protein source, functional properties are just as important as nutritional quality. For this reason, Patil and Benjakul [141] conducted a comparative analysis of albumin and globulin fractions extracted from defatted coconut meat. Their study revealed differences in amino acid profiles and molecular weights, with the dominant protein at 55 kDa and glutamine and glutamic acid as major amino acids in both fractions. Notably, the globulin fraction exhibited higher surface hydrophobicity and greater susceptibility to alcalase hydrolysis. Emulsions stabilized with globulin demonstrated superior performance, exhibiting smaller oil droplet sizes, reduced coalescence index, and lower flocculation factor compared to those stabilized with albumin. Further studies have examined how centrifugally separated protein fractions from virgin coconut oil differ in structure and functionality [142]. Glutamic acid was the most prevalent amino acid in aqueous and solid-phase fractions, but the essential amino acid content was 33.8% lower in aqueous-phase proteins than in solid-phase proteins. Aqueous-phase proteins exhibited greater structural disorder, solubility, foaming capacity, and emulsifying stability, while solid-phase proteins demonstrated superior water holding capacity. These insights support the broader potential for valorizing coconut oil by-products as sustainable, functional ingredients in the food industry.

The processing effect is another important parameter for the quality and functionality of proteins. Liu et al. [143] investigated the impact of dry processing on coconut protein isolates. Their findings revealed that drying altered the amino acid composition, with essential amino acids accounting for 37.87% and 37.21% of the total amino acids in proteins derived from defatted copra meal and defatted desiccated copra meal, respectively. Additionally, dry processing resulted in reduced surface hydrophobicity, total and free sulfhydryl content, solubility, and free amino acid levels. In contrast, improvements were observed in water holding capacity, oil holding capacity, and foam stability. In a related study, sequential extraction was used to isolate albumin, globulin, prolamin, glutelin-1, and glutelin-2 fractions from coconut cake, and their antioxidant activities were assessed [144]. All fractions, except albumin, demonstrated strong radical-scavenging and ion-chelating capacities. Moreover, all fractions except glutelin-2 provided protection against oxidative DNA damage, highlighting their potential as natural antioxidants in functional food formulations. These studies collectively support the valorization of coconut oil by-products as promising protein-rich ingredients for nutritional enhancement.

Coconut oil by-products also represent a valuable and sustainable source of dietary fiber. Analysis of coconut flour obtained from virgin coconut oil residue showed high fiber content, including 38.0% total dietary fiber, 38.3% neutral detergent fiber, 24.4% acid detergent fiber, 14.1% hemicellulose, and 10.3% cellulose [145]. Recent research has explored the effects of various extraction techniques on the physicochemical and functional properties of dietary fiber. For example, three extraction methods—enzymatic-chemical, subcritical water, and ultrasonic-chemical—were applied to defatted coconut flour to obtain soluble dietary fiber [146]. Subcritical water extraction yielded the highest recovery (13.99 ± 0.12 g/100 g) and produced fibers with more porous microstructures, lower molecular weights, enhanced gelling ability, higher thermal stability, and reduced crystallinity. Mannose (162.79–167.07 mg/g) and galactose (11.97–68.66 mg/g) were the dominant monosaccharides. This method also led to superior water/oil holding capacities and increased glucose adsorption, pancreatic lipase inhibition, cholesterol adsorption, and nitrite ion binding. Another study assessed the influence of acid treatment, cellulase hydrolysis, particle size reduction, and pH on the adsorption capacity of defatted coconut cake fibers [147]. Cellulase hydrolysis significantly improved soluble fiber content, water holding capacity, and the adsorption of cholesterol, bile acids, and nitrites. Acid treatment further enhanced oil holding and adsorption capacities. Reducing particle size from 250 to 167 µm and lowering the pH from 7.0 to 2.0 both increased adsorption performance.

Yan et al. [148] examined the impact of steam explosion and extrusion on the structural and functional properties of dried coconut dietary fiber. These treatments increased the soluble fiber content by 115% and 229%, respectively, compared to the untreated control. Water holding capacity improved from 8.51 to 11.81 and 12.47 g/g; oil holding capacity from 5.42 to 8.30 and 9.91 g/g; and water-swelling capacity from 8.00 to 12.50 and 15.34 mL/g, respectively. Both treatments also enhanced thermal stability and functional parameters such as glucose and cholesterol adsorption, *α*-amylase inhibition, cation exchange capacity, and nitrite binding. Among the methods tested, extrusion was deemed the most effective and offers strong potential for application in food industry formulations. Given its affordability and health-promoting properties—such as antidiabetic, cardioprotective, prebiotic, immunomodulatory, and anti-obesity effects—coconut flour is a promising functional ingredient.

Coconut oil by-products have shown potential as natural antioxidants. However, compared to fiber and protein, fewer studies have explored their phenolic content and antioxidant properties. Kasapoğlu et al. [149] reported that cold-pressed coconut oil by-products contained 104.48 mg GAE/100 g of total phenolics and 106.58 mg TE/100 g antioxidant capacity (via CUPRAC assay), indicating notable bioactive potential. Further analysis of these by-products revealed that gallic acid was the predominant phenolic compound (386.28 mg/100 g), followed by caffeic, ferulic, *p*-hydroxybenzoic, *p*-coumaric, and syringic acids [150]. The total phenolic content was 67.66 mg GAE/100 g, with a CUPRAC activity of 106.32 mg TE/100 g and a phenolic recovery of 12.16%. The extracts also demonstrated antibacterial activity, with inhibition zones ranging from 6.83 to 8.50 mm against various gram-positive and gram-negative bacteria.

Seneviratne et al. [151] identified several phenolic acids in ethanolic extracts of coconut oil cake, including chlorogenic acid (240.8 ± 5.4 mg/kg), caffeic acid (225.7 ± 82.2 mg/kg), and *p*-hydroxybenzoic acid (129.0 ± 6.8 mg/kg), along with flavonoids such as apigenin (56.7 ± 9.8 mg/kg) and catechin (26.9 ± 6.4 mg/kg). Compared to synthetic antioxidants, the coconut oil cake extract showed better color retention (96 ± 2%) than butylated hydroxytoluene (BHT) (89 ± 2%) in a *β*-carotene-linoleate model at 60 µg/mL over two hours. Thermal stability followed the order: TBHQ > the extract > BHA > BHT. These findings underscore the multifunctional potential of coconut oil processing by-products as sustainable sources of protein, dietary fiber, and natural antioxidants for diverse food applications.

Coconut oil by-products have attracted interest due to their richness in valuable compounds. In addition, coconut and its derivatives—such as coconut milk, water, oil, and flour—are already widely used in bakery and pastry products that are popular among consumers. As a result, most studies explore how coconut processing residues can be used in such food products. For instance, Fonseca-Bustos et al. [137] reformulated baked snacks by incorporating coconut flour, a by-product of virgin coconut oil production, to improve their nutritional profile. Using a Box–Behnken design, the optimal formulation conditions were determined as 55.3% coconut flour, a baking time of 20 min, and a temperature of 159 °C. Compared to nixtamalized corn flour, the addition of coconut flour enhanced protein, fat, and ash content in the final product. Lauric acid, a characteristic fatty acid of coconut, was identified as the predominant fatty acid, alongside others such as caproic, caprylic, capric, and myristic acids.

White rolls have also been investigated for the incorporation of coconut oil residues [152]. The effects on nutritional composition, sensory quality, physical attributes, and texture were assessed. The addition of these residues improved roll yield and crumb moisture and reduced baking loss. However, some adverse effects were observed, including reduced roll volume and increased hardness and chewiness. Notably, the sensory evaluation revealed no negative perception up to a 12% addition level, with the fortified rolls being well accepted by consumers. At this level, protein and dietary fiber increased by 7.9% and 76%, respectively, while caloric content was reduced from 265.7 to 244.6 kcal/100 g compared to control samples.

In another study, Sarabandi et al. [153] explored the reformulation of bread by incorporating peptides obtained from enzymatically hydrolyzed coconut meal protein. These peptides were encapsulated using spray-drying with maltodextrin and maltodextrin–pectin blend to stabilize them and mask their bitterness. Antioxidant assays (DPPH and ABTS) showed enhanced antioxidant activity in the fortified bread, particularly when the maltodextrin–pectin carrier was used. The improved antioxidant capacity was attributed to better thermal protection of bioactive peptides during baking. Furthermore, the encapsulation system effectively masked the bitterness, supporting the sensory quality of the final product while boosting its functionality.

Additionally, the potential of cold-pressed coconut oil by-products in plant-based beverages and low-fat ice cream was assessed [149]. In the first phase, a plant-based coconut drink made from these by-products was compared to a commercial equivalent. The experimental drink exhibited higher fat and protein content, higher zeta potential, and smaller particle size, with physical stability levels of 98%, comparable to the commercial product’s 99%. These findings confirmed the viability of using coconut by-products for plant-based drink production. In the second phase, full-fat and low-fat ice cream samples were prepared using the formulated coconut drink and compared with controls based on commercial coconut beverages. All ice cream variants showed desirable rheological properties (such as shear-thinning behavior, solid-like structure, and recovery after deformation). Importantly, the use of coconut by-product-based drinks did not negatively impact sensory quality, supporting their feasibility as a cost-effective raw material in both full-fat and reduced-fat plant-based ice cream formulations.

Further recent studies on the valorization of coconut oil processing by-products as functional food ingredients are summarized in Table 3.

Overall, it is evident that the waste and by-products resulting from the production of oil from oily fruits constitute a substantial reservoir of valuable bioactive compounds, particularly with regard to human health and well-being. However, in order to obtain these health-promoting compounds and incorporate them into food products on a large scale, extraction, purification, and innovative and eco-friendly technologies must be understood in detail and optimized. Moreover, further research is required to ascertain the potential health benefits of fortified foods. It is imperative that this must include further toxicity assays, as well as in vitro and in vivo studies for the designing of new functional foods. This additional research may be more successful in raising consumer awareness and adjusting these fortified foods to human consumption patterns.

## 3. Valorization of Waste and By-Products from Oilseed-Based Oil Processing

**Figure 5:**
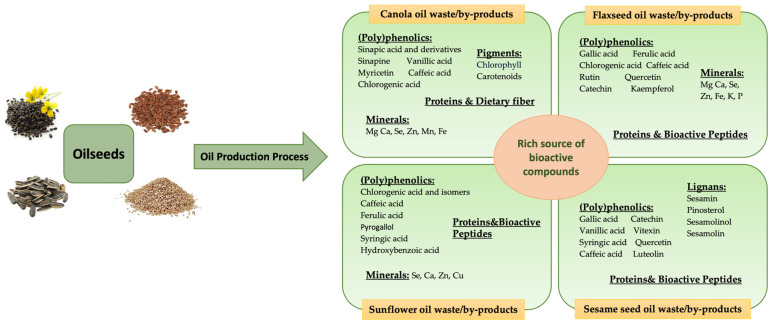
Bioactive potential of oilseed-based oil processing waste/by-products.

Oilseeds (such as canola/rapeseed, sunflower, flaxseed, and sesame) are cultivated worldwide for their edible oils. However, their processing yields substantial quantities of by-products that are often underutilized, despite having rich nutritional and functional profiles (Figure 5). Reintegrating these by-products into the functional food formulations supports clean-label product development and contributes to global sustainability goals. Therefore, oilseed by-products represent a valuable opportunity for creating health-promoting ingredients from materials traditionally regarded as waste.

### 3.1. Canola/Rapeseed Oil Processing Waste/By-Products

In 2022, global canola/rapeseed oil production reached approximately 26.69 million tons. This oil, mainly extracted from *Brassica napus* L., *Brassica rapa* L., and *Brassica juncea* L., ranked third in global vegetable oil production after palm and soybean oils. The major producers were Germany (3.72 million tons), Canada (3.65 million tons), China (3.62 million tons), and India (3.45 million tons). Regionally, Europe accounted for the largest share of production (44.4%), followed by Asia (34.4%) and the Americas (19%).

This large-scale production generates significant quantities of by-products, such as rapeseed meal, which is rich in bioactive and functional compounds [6]. Compared to whole seeds, rapeseed meal—especially from cold-pressing—has richer bioactive composition and higher levels of protein (35.04%), fiber, ash, and carbohydrates, and minerals such as magnesium, calcium, selenium, zinc, manganese, and iron [160]. In contrast, rapeseed hulls constitute about 17% of the seed’s total weight and are a good source of dietary fiber. However, they also contain anti-nutritional compounds such as glucosinolates, waxes, phytic acid, and pigments. Notably, dehulling before pressing improves oil quality and increases the protein content of the resulting cake by up to 40% [161]. These findings highlight the importance of preprocessing steps, which influence not only oil quality but also the composition and value of the resulting by-products.

Rapeseed meal is particularly valued for its protein content, predominantly consisting of the storage proteins cruciferin and napin. These proteins have demonstrated potential health benefits, especially in the management of hypertension, diabetes, and oxidative-stress-related conditions [162]. The amino acid profile of rapeseed meal is relatively balanced. It is especially rich in glutamate + glutamine (up to 14.03%) and aspartate + asparagine (up to 12.5%), and it also provides a notable amount of sulfur-containing amino acids such as methionine + cysteine (up to 2.89%). Although lysine and tryptophan are present at moderate levels, they still contribute to the overall nutritional value [23,160]. Thus, exploring green and sustainable extraction techniques is essential for utilizing protein-rich by-products like rapeseed meal to meet the growing demand for plant-based proteins. For example, James and Euston [163] investigated the use of NADES composed of betaine/citric acid and choline chloride/glycerol mixtures to extract protein from rapeseed press cake. These NADES formulations significantly improved protein yield compared to both water and alkaline extraction. Molecular dynamics simulations further suggested that glycerol–choline chloride NADES can disrupt protein–water interactions, potentially enhancing extraction efficiency.

Beyond its macronutrient profile, rapeseed meal is also a valuable source of phenolic compounds. Multescu et al. [164] reported that rapeseed meal contains a total phenolic content of 11.24 ± 0.65 mg GAE/g and a total flavonoid content of 9.46 ± 0.50 mg QE/g, with flavonoids accounting for approximately 84.16% of total polyphenols. However, higher values, up to 63.95 mg GAE/g, have been reported, depending on extraction conditions. Rapeseed meal showed strong antioxidant capacity via the CUPRAC method, which effectively measures the total reducing capacity of hydrophilic and lipophilic antioxidants. Among the tested methods (DPPH, ABTS, FRAP, and CUPRAC), CUPRAC values showed the strongest positive correlation with TPC. When evaluating antioxidant capacity in oily and protein-rich by-products, TAC assays should measure both hydrophilic and lipophilic antioxidants and also respond to protein thiols. For example, the CUPRAC reagent includes a hydrophilic Cu(II) core and strongly bound hydrophobic neocuproine ligands, allowing it to measure antioxidants regardless of their polarity. Additionally, the CUPRAC method can oxidize protein thiols to disulfides in a well-defined stoichiometric reaction in urea buffer [165]. Similarly, Rabiej-Kozioł and Szydłowska-Czerniak [166] confirmed that the CUPRAC assay is a highly effective method in determining the antioxidant activity of black cumin oil and its by-products, due to the lipophilic and hydrophilic nature of their antioxidants.

The predominant phenolic acid is sinapic acid and its derivatives, which comprise 73–99% of total phenolics. These are mainly present in esterified forms such as sinapine and glycosides like 1-*O*-β-D-glucopyranosyl sinapate [23]. Hussain et al. [167] also identified that canola meal mainly contains hydroxycinnamic acid derivatives, with sinapine and trans-sinapic acid being the most abundant and primarily responsible for its antioxidant and radical scavenging activity. Kaempferol conjugated with sinapic acid was also identified. Furthermore, Petraru and Amariei [160] quantified the total phenolic content in rapeseed meal at 9272 mg/kg, with major contributors including vanillic acid, myricetin, chlorogenic acid, and caffeic acid, reinforcing the meal’s compositional richness.

Table 4 below summarizes various fortified foods incorporating rapeseed/canola oil waste or by-products, detailing the form of additive used, the food products involved, and the resulting quality attributes.

Several studies have explored how rapeseed-derived phenolic compounds improve the oxidative stability of plant-based oils during storage and heating. A notable method involves using ethanolic washout fractions from rapeseed meal protein extraction. These fractions are particularly rich in simple phenolic compounds such as hydroxybenzoic acid (179.35 ± 43.01 mg/L) and protocatechuic acid (2.27 ± 0.31 mg/L) [168]. The Rancimat method was used to assess the oxidative stability of soybean, rapeseed, and sunflower oils enriched with 5% and 15% ethanol-based fractions. Specifically, the addition of a 15% ethanol-soluble fraction to rapeseed oil resulted in a higher antioxidant activity (1.18) compared to 0.02% BHT (1.09). This concentration was also more effective than adding a 5% equivalent addition [168]. Researchers have also explored encapsulation to enhance the stability and function of rapeseed-derived phenolics. Zadbashkhanshir et al. [169] identified maltodextrin-based encapsulation followed by freeze-drying as the most efficient method. When these encapsulated phenolics were added to canola oil at 200, 400, and 800 ppm, they exhibited oxidative protection comparable to 200 ppm of TBHQ, a synthetic antioxidant. Rabiej-Kozioł and Szydłowska-Czerniak [170] showed that acid-hydrolyzed, freeze-dried rapeseed meal extracts significantly enhanced TPC and antioxidant activity in refined rapeseed oil. This effect was observed during 24 h of deep-frying French fries. Oils enriched with the extract showed markedly lower peroxide value, *p*-anisidine value, and total and integral oxidation scores (TOTOX, and INTOX) compared to controls, confirming their ability to reduce thermal oxidation and extend shelf life.

Recent studies also show that rapeseed cake can boost antioxidant activity in different food systems. For instance, microwave pretreatment (optimal: 800 W, 3 s) significantly improved the release of bioactive compounds in plant-based milk alternatives formulated with rapeseed cake and black cumin cake. The rapeseed-based milk contained higher levels of lipophilic antioxidants such as tocopherols, chlorophylls, and carotenoids. In contrast, the black cumin formulation was richer in phenolic acids [171]. Another study found that rapeseed press cake had the highest antioxidant capacity among tested ingredients (wheat flour, rapeseed oil, margarine, coconut oil). Incorporating it in biscuits enhanced antioxidant activity. However, excessive amounts negatively affected sensory quality. This finding highlights the need for careful formulation when using rapeseed press cake as a functional ingredient [172].

Rapeseed by-products show promising bioactive potential, yet key gaps remain. Limited data exist on the bioaccessibility and bioefficacy of proteins and phenolics in real food matrices. Most antioxidant assessments rely on in vitro assays, lacking physiological relevance. The scalability and regulatory acceptance of green extraction methods (e.g., NADES) require further validation. Additionally, the impact of rapeseed-derived compounds on sensory properties and stability in food systems remains underexplored.

### 3.2. Sunflower Oil Processing Waste/By-Products

According to FAOSTAT data, global sunflower (*Helianthus annuus*) oil production reached approximately 20.26 million tons in 2022, ranking fourth among plant-based oils after palm, soybean, and rapeseed. Major producers include Russia (5.99 Mt), Ukraine (4.58 Mt), Argentina (1.50 Mt), Turkey (0.90 Mt), Bulgaria (0.83 Mt), and Hungary (0.73 Mt), with Europe accounting for 75.3% of global output, followed by Asia (10.6%) and the Americas (10%). This large-scale production generates substantial amounts of by-products, particularly sunflower meal, offering opportunities for valorization in both food and non-food sectors [6].

Sunflower meal, mostly obtained from oilseed varieties (about 90% of production), is a protein-rich by-product. It contains 40–50% protein, and bioactives like helianthinin, albumins, and sulfur-containing amino acids (e.g., methionine, cysteine) [173]. Oil extraction increases the concentration of essential minerals like selenium, calcium, zinc, and copper in the cake while reducing them in the oil phase. The meal is also a good source of unsaturated fatty acids (oleic and linoleic acids) and essential amino acids—particularly valine, tryptophan, and isoleucine—making it a promising ingredient for functional food formulations [174].

Sunflower by-products also contain 3–7% phenolic compounds. Chlorogenic and caffeic acids together account for up to 73% of the total phenolics [175,176,177]. The broader phenolic profile includes dicaffeoylquinic, feruloylquinic, and coumaroylquinic acids. Among them, chlorogenic acid isomers (3-, 4-, and 5-O-caffeoylquinic acids) are particularly dominant [175,176]. Notably, chlorogenic isomers alone account for up to 78% of total phenolics. Studies show that sunflower meal has a more diverse phenolic profile than rapeseed meal. It includes compounds like pyrogallol, syringic acid, and hydroxybenzoic acid [168]. Multescu et al. [164] reported that sunflower meal exhibited a total phenolic content of 11.70 ± 0.35 mg GAE/g and a total flavonoid content of 7.92 ± 0.14 mg QE/g, with flavonoids comprising about 67.69% of the total phenolics. The predominant phenolic compounds in sunflower meal include chlorogenic acid and caffeic acid, which together represent nearly 70% of its phenolic composition. In terms of antioxidant potential, sunflower meal demonstrated notable activity across all tested assays, with the CUPRAC assay showing a particularly strong correlation with TPC. This suggests that the phenolic compounds in sunflower meal, especially chlorogenic acid derivatives with multiple hydroxyl groups, significantly contribute to its reducing power as detected by the CUPRAC method.

Several factors influence the phenolic content of sunflower meal. These include genotype, growing conditions (soil, water, climate), and processing steps such as dehulling and oil extraction. While total composition may not change drastically, dehulling and de-oiling significantly affect the fiber, protein, and phenolic profiles [177]. Generally, partially hulled seeds yield the highest phenolic concentrations, whereas unhulled samples exhibit lower phenolic levels, likely due to phenolic–lignocellulose interactions in the hull [176]. For instance, unhulled micronized sunflower meal has been shown to contain 14.18 mg/g chlorogenic acid. This compound accounts for approximately 86% of the TPC [178].

Table 5 presents an overview of fortified food products enriched with sunflower oil waste/by-products, highlighting the type of additives applied and their impact on the final product quality.

Several studies have explored the incorporation of sunflower meal in food products. Adding 5–15% sunflower seed cake flour to gluten-free bread increased polyphenol content by up to 2.5 times and improved crumb color and taste. However, levels above 15% reduced volume and springiness by disrupting dough structure [181]. Similarly, the addition of defatted sunflower flour (2–4%) to frankfurters enhanced polyphenol content, mainly due to caffeic and ferulic acids [182].

However, phenolic compounds can complicate protein extraction by forming insoluble complexes with amino acids such as lysine and methionine, resulting in browning and reduced protein digestibility [177]. Francesca et al. [179] found that sunflower protein concentrates had the highest phenolic content (35.20 mg GAE/g) compared to whey and pea protein samples, mainly due to their chlorogenic acid. Nevertheless, sunflower protein dispersions showed an 84.3% reduction in phenolic content after in vitro digestion, suggesting low bioaccessibility, possibly due to gastrointestinal instability and interactions with other matrix components.

Innovative extraction strategies like NADES have shown promise in overcoming such limitations. Şen et al. [16] reported that NADES-extracted sunflower pomace had significantly higher antioxidant activity and phenolic content than ethanol or methanol extracts. When incorporated into smoothie-like beverages, these extracts enhanced antioxidant activity across all digestion stages, particularly at 20% enrichment. The NADES matrix likely stabilized phenolics through hydrogen bonding and protection during digestion. In another study, de Sousa Bezerra et al. [183] assessed the extraction efficiency and stability of phenolic compounds from sunflower meal using various NADES formulations. The most effective solvent was a NADES mixture of lactic acid and glucose (5:1), which yielded 1786 mg/L of chlorogenic acid. Superior protection against both heat treatment (60 °C) and storage (30 days) was also observed with the use of the acidic NADES. de Sousa Bezerra et al. [184] also found that acetonitrile was the most effective solvent for extracting phenolics from sunflower meal using choline chloride:glycerol and urea:glycerol NADES. Recovery rates were 80% and 63%, respectively. These NADES combinations resulted in a significant reduction in cell viability, with a maximum decrease of 78.4% observed in MCF-7 cells and 74% in MDA-MB-231 cells.

While phenolic–protein interactions can hinder digestibility and nutrient absorption by inhibiting enzymes like trypsin, chymotrypsin, and lipase [173], targeted removal or recovery of phenolics can improve protein usability. Although complete dephenolization is challenging, partial removal enhances protein extraction, digestibility, and functional properties. Recovered phenolics can further be repurposed as natural antioxidants in food or nutraceuticals [177]. For instance, ethanol washout fractions from sunflower meal protein extraction (15%) provided superior oxidative protection in sunflower and soybean oils, comparable to that of synthetic antioxidant BHT (0.02%) as measured by Rancimat [168]. Moreover, Michalska-Ciechanowska et al. [180] showed that adding sunflower seed cake washouts to whey significantly increased both total phenolic content and antioxidant capacity. At ≥40–50% concentrations, strong antioxidant effects were linked to chlorogenic, neochlorogenic, and caffeic acids. However, higher levels also decreased free amino groups and changed protein profiles, suggesting phenolic–protein conjugation.

Sunflower processing by-products, particularly oilcake and meal, offer strong potential for functional food applications due to their richness in phenolic compounds, proteins, and bioactive constituents. However, the presence of phenolics can negatively affect protein digestibility and functionality, highlighting the need for optimized extraction and purification strategies. Valorization of these compounds in both food and non-food sectors offers a sustainable pathway to develop high-value products and reduce industrial waste.

Despite the recognized nutritional and functional potential of sunflower by-products, key research gaps persist. The impact of phenolic–protein interactions on digestibility and functional performance remains underexplored, particularly in complex food matrices. Furthermore, while NADES-based extractions show promise, their scalability, regulatory acceptance, and compatibility with food applications require further validation. Standardized methods are also needed to assess antioxidant activity and phenolic retention across processing stages. Future research should focus on optimizing extraction–purification strategies, improving bioaccessibility, and evaluating techno-functional and sensory impacts in real food systems.

### 3.3. Flaxseed Oil Processing Waste/By-Products

Flaxseed (*Linum usitatissimum* L.) oil contributed approximately 730 thousand tons, representing roughly 0.3% of the global output in 2022 [6]. Flaxseed meal, the by-product remaining after oil extraction, typically contains 20–35% protein, 2–40% fat, 28–37% dietary fiber, 5–6% moisture, and up to 5.5% ash, along with essential minerals and vitamins. Additionally, it is rich in bioactive compounds such as lignans (0.3–0.9%), phenolic compounds, and alkaloids, which contribute to its potent antioxidant activity [185,186,187,188,189].

Flaxseed cake contains a wide range of phenolic compounds, including major phenolic acids (gallic, ferulic, chlorogenic, caffeic, and protocatechuic acids), and flavonoids like rutin, quercetin, catechin, and kaempferol [186,189,190]. Treatments, such as microwave heating and ultrasound, have been shown to enhance total phenolic content and antioxidant potential, thereby increasing the functional value of flaxseed cake [189,191].

Among lignans, secoisolariciresinol diglucoside is the dominant compound in flaxseed products, known for its potent antioxidant and anti-inflammatory properties [190,192]. Secoisolariciresinol diglucoside and other phenolics are mostly bound within the flaxseed matrix. However, processes like steam explosion, extrusion, and fermentation can effectively release them. Likewise, flavonoids (e.g., quercetin and dihydroquercetin) and phenolic acids (e.g., ferulic and erucic acids) are released through cell wall breakdown by mechanical, thermal, or enzymatic methods. This enhanced release results in significant increases in total phenolic content and antioxidant activity [185,193].

Flaxseed cake also possesses a distinctive amino acid profile, characterized by notably high levels of glutamic acid, arginine, valine, threonine, and serine, as well as essential amino acids like methionine [188,194]. Compared to soybean, sunflower, peanut, and pumpkin cakes, flaxseed cakes contain lower amounts of histidine and arginine [194]. Building on this favorable amino acid composition, enzymatic hydrolysis of flaxseed proteins—particularly when combined with ultrasound or microwave pretreatment—has been shown to enhance the production of bioactive peptides with strong antioxidant potential [195,196,197]. For example, Wei et al. [197] showed that ultrasound-assisted hydrolysis with alkaline protease released more acidic (Glu, Asp) and basic amino acids (Arg, Lys, His) and identified three antioxidant peptides (PFFWLHHT, HCLEFLSPRF, ALTMPHNW) with strong DPPH radical-scavenging activity.

Furthermore, flaxseed cake is a valuable source of essential minerals, including potassium, phosphorus, magnesium, calcium, iron, zinc, copper, and selenium [186,189]. Some processing methods, such as microwave treatment, have been shown to enhance the levels of calcium, sodium, iron, zinc, and copper [189]. Adding 5% flaxseed meal to baked products like toast and cake significantly boosted minerals such as potassium, magnesium, phosphorus, and calcium. This shows its potential to enhance wheat-based products nutritionally [186].

As shown in Table 6, different forms of flaxseed oil waste/by-products have been utilized as ingredients in food fortification, influencing the overall quality of the finished products.

Flaxseed cake is rich in antioxidants, polyunsaturated fatty acids, and essential amino acids. This makes it a valuable functional ingredient in bakery products. Multiple studies have shown that adding flaxseed cake to foods, especially bread, enhances total phenolic content, antioxidant capacity, and nutritional quality in a dose-dependent manner. This includes increased levels of n-3 polyunsaturated fatty acids [187,192,198,199]. Specifically, Krupa-Kozak et al. [200] showed that, in addition to increases in total phenolic content and antioxidant capacity, flaxseed oil cake enriched protein and mineral contents (notably potassium, magnesium, and zinc). They also noted that flaxseed cake components may enhance the formation of Maillard reaction products, particularly melanoidins during baking. These compounds boost antioxidant capacity, especially in the bread crust [187,199,200].

The incorporation of flaxseed into fermented food systems—such as kefir-like beverages and plant-based Camembert analogs—has been shown to enhance their nutritional and functional properties through microbial activity. Łopusiewicz et al. [201,202] found that fermentation significantly increased total polyphenol and total flavonoid contents. This was mainly due to microbial enzymatic hydrolysis of polysaccharides, which release bound phenolics. These changes led to stronger antioxidant activity, linked to the formation of bioactive peptides and microbial biotransformation of phenolic compounds. In the kefir-like beverage study, strong antioxidant capacity was maintained during 21 days of storage at 6 °C despite some post-fermentation fluctuations. Similarly, in the Camembert analog study [202], both oxidative stability and antioxidant capacity were maintained throughout 28 days of refrigerated storage at 6 °C.

Regarding a different product, mortadella, Pinton et al. [188] reported that incorporating flaxseed cake at 1.5% and 3% levels in place of alkaline phosphate led to increased lipid oxidation due to the bioactive compounds present in flaxseed cake. However, TBARS values stayed below sensory thresholds during storage. This suggests that flaxseed cake can replace phosphate, though extra antioxidant strategies may be needed to ensure oxidative stability.

The low bioaccessibility and matrix-bound nature of phenolics and lignans—especially secoisolariciresinol diglucoside—limit their bioefficacy, necessitating further optimization of release strategies using enzymatic, thermal, or microbial treatments. Additionally, while antioxidant peptides have been identified, their structure–function relationships, stability during digestion, and in vivo bioactivities are poorly understood. Moreover, the dual role of flaxseed bioactives in enhancing nutritional quality yet promoting lipid oxidation in meat products highlights the need for tailored antioxidant systems in specific food matrices. Comprehensive techno-functional and safety evaluations are essential to support broader applications of flaxseed cake in diverse food systems.

### 3.4. Sesame Seed Oil Processing Waste/By-Products

In 2022, about 200 million tons of crude plant-based oil were produced worldwide. Sesame seed (*Sesamum indicum*) oil made up about 1.0 million tons, or roughly 0.5% of this total [6]. The main by-product of sesame oil extraction, sesame cake, is a nutrient-dense matrix that holds considerable potential for valorization in food systems. The composition of sesame cake varies widely. Moisture content ranges from 4.26% to 7.7%, ash from 3.64% to 13.06%, fat from 14.33% to 49.55%, protein from 31.68% to 58.7%, carbohydrates from 19.07% to 36.64%, and fiber from 5.04% to 10.35% [203,204,205,206,207].

In terms of phenolic composition, defatted sesame cake is particularly rich in both free and bound phenolics. Sundar et al. [208] identified several hydroxybenzoic acids in sesame cake, including gallic, protocatechuic, 4-hydroxybenzoic, vanillic, and syringic acids. They also found many hydroxycinnamic acids, with rosmarinic acid. They also detected various flavonoids, such as catechin, vitexin, quercetin, naringenin, luteolin, and apigenin. Levels of 4-hydroxybenzoic acid and total hydroxycinnamic acids were notably high. Complementarily, Lucini Mas et al. [209] reported that the dominant phenolics in defatted sesame flour are lignans, particularly sesaminol derivatives, which account for about 60% of the total lignan content. Other relevant lignans include pinoresinol, sesaminol, sesamolinol, and their glycosylated or acetylated derivatives. These compounds are sensitive to processing. For example, roasting can reduce some lignans but increase the extractability of others. Furthermore, sesame cake contains greater amounts of lignans (13.4 mg/g) and tocopherols (430 µg/g) than the seed itself, supporting its strong antioxidant potential [205]. In addition, mineral analysis conducted by El-Enzi et al. [206] revealed that sesame cake flour is rich in essential minerals including calcium (1200.02 mg/100 g), magnesium (185.71 mg/100 g), zinc (3.8 mg/100 g), iron (10.6 mg/100 g), phosphorus (580.52 mg/100 g), and potassium (374.71 mg/100 g).

Sesame oil cake and its protein fractions provide a rich source of essential amino acids (e.g., leucine, methionine, lysine). They also contain non-essential amino acids with antioxidant properties (e.g., glutamic acid, aspartic acid, arginine) [206,210]. Albumin and glutelin fractions were especially abundant in threonine, proline, and tyrosine, while prolamin was notable for its high content of hydrophobic amino acids. Functionally, the albumin fraction demonstrated the strongest hydroxyl radical-scavenging activity, followed by weaker effects from glutelin and prolamin. In support of these findings, Norouzi et al. [211] demonstrated that enzymatic hydrolysis of protein isolates (~89% protein) with alcalase, flavourzyme, or trypsin increased antioxidant activity significantly. Alcalase was the most effective, achieving the highest degree of hydrolysis and antioxidant capacity after 4 h by releasing bioactive peptides and amino acids. Similarly, Noptana et al. [204] found that hydrolysates obtained using Subtilisin A from *Bacillus licheniformis* exhibited strong antioxidant effects, particularly in peptide fractions <1 kDa, which benefited from greater solubility, enhanced exposure of active residues, and potential synergy with sesame polyphenols. Comparing traditional alkali-soluble acid precipitation with NADES solvents for protein extraction from sesame meal showed that choline chloride–ethylene glycol DES extraction yielded purer protein (up to 93%) than the traditional method (77.1%). A subsequent comparison of the functional properties of these proteins indicated that choline chloride–oxalic acid DES-extracted protein exhibited particular advantages, especially in terms of solubility, which could reach 61.06% [212]. These results collectively demonstrate the high bioactive potential of sesame-derived proteins and peptides as natural antioxidants in food and health applications.

A list of foods fortified with sesame seed oil waste/by-products, including the additive forms and quality outcomes, is compiled in Table 7.

Besides its biochemical richness, sesame oil cake has practical uses as a functional ingredient in many food products. Gaipova et al. [205] found that adding sesame cake powder to mayonnaise slowed lipid oxidation during 90 days of storage at 60 °C. While some sensory decline occurred over time, fortified samples kept better color, texture, and overall acceptability than controls. Likewise, Sengupta et al. [213] reported that yogurt made from defatted sesame cake suspension enriched with rice bran oil had higher total phenolic and flavonoid content and antioxidant activity than yogurt made with whole sesame seeds. These effects increased with higher defatted sesame cake levels (6–8%).

Sesame oil cake use in bakery products is also growing because it improves nutritional and functional qualities. Many studies report increased phenolic content and antioxidant activity, mainly due to lignans and polyphenols. For instance, Yaver et al. [216] observed a dose-dependent increase in antioxidant properties of gluten-free crackers enriched with defatted sesame meal, with the highest values seen at 20% inclusion. El-Enzi et al. [206] demonstrated that replacing wheat flour with 20–40% sesame cake flour in biscuits significantly improved protein, fiber, fat, ash, and mineral content—particularly calcium, iron, and zinc—while reducing carbohydrate levels. Mohammady Assous et al. [207] reported that biscuits made with a filling containing 80% date paste and 20% sesame cake exhibited superior nutritional quality, with elevated levels of protein, essential amino acids, polyphenols, and minerals, making them promising for school nutrition programs. Further supporting these benefits, Lucini Mas et al. [209] showed that adding defatted sesame flour to cookies tripled total phenolic content and boosted antioxidant capacity. Processing-induced changes such as Maillard reactions also influenced the phenolic profile. Digestion lowered total polyphenol recovery, but a significant portion remained bioavailable in the colon, suggesting prebiotic and health benefits. In a related study, Nouska et al. [215] enriched wheat bread with low-fat and high-fat sesame cake (6–20%), which significantly increased lignan (e.g., sesaminol triglucoside, sesaminol diglucoside, sesamolin) and phenolic acid (e.g., ferulic, caffeic, *p*-coumaric) content. Despite partial degradation of sesaminol derivatives during baking, fortified breads maintained superior antioxidant capacity and bioactive density compared to controls. Collectively, these results highlight sesame cake’s value as a functional ingredient for enhancing the health-promoting potential of baked goods.

Beyond bakery applications, sesame-derived ingredients also improve the quality of plant-based meat analogs. Sundar et al. [208] found that adding defatted sesame cake powder to meat analogs increased free and bound phenolic compounds. Levels of *trans*-cinnamic acid and hydroxycinnamic acids rose with increasing sesame cake content. Catechin was the predominant flavonoid, while bound forms of vitexin and quercetin also increased. Additionally, flavonoids such as naringenin and luteolin, which were originally low or absent in the base matrix, were substantially enriched. These results show sesame cake amount mainly raised phenolic levels, with a minor effect from processing factors like feed moisture. Overall, these findings underscore the potential of sesame-based ingredients to improve the antioxidant profile of diverse food systems.

The structure–function relationships, gastrointestinal stability, and in vivo bioactivities of bioactive peptides derived from sesame proteins need deeper investigation. Moreover, the long-term effects of sesame cake incorporation on sensory attributes and oxidative stability in diverse food products, including meat analogs and bakery items, are not fully understood. Future research should also explore optimized extraction techniques and synergistic antioxidant strategies to maximize the functional benefits while ensuring product quality and consumer acceptance.

## 4. Limitations and Future Perspectives

Waste and by-products from the plant-based oil industry are a considerable source of nutritious and bioactive compounds offering potential health benefits. They can be used in a variety of applications in the creation of functional food formulations, representing a significant opportunity for the food industry and offering a potential solution to economic and environmental issues, as well as providing possible reduction in malnutrition and poverty worldwide. This is also an ideal chance to meet consumers’ growing demand for healthier, more natural foods. The process of food fortification can be achieved through the direct addition of produced oil waste/by-products or the incorporation of extracts of high-value compounds, including dietary fiber, protein, and phytochemicals, into the formulation. At this stage, one of the most critical issues is ensuring the safety of oil waste and by-products for consumption. Due to their individual properties and processing methods, they can contain toxins and potential pathogenic microorganisms or chemical contaminants. For example, the quality and safety of several plant-based oil industry by-products were investigated by microbiological tests and mycotoxin assessment [217]. The levels of spoilage microorganisms (i.e., molds) were found to be lower than the established limits for comparable matrices, as determined by microbial analysis. Conversely, a moderate aerobic microbial contamination was detected. Furthermore, deoxynivalenol contamination was reported, but it was indicated that the concentrations of aflatoxin in the samples were below the limits of detection of the enzyme-linked immunosorbent assay (ELISA) with regard to the total aflatoxin/mycotoxins. Thus, it is vital to ensure that they are free from any harmful compounds before considering them as food ingredients. Storage conditions are also very significant for microbial safety, especially during long storage periods, due to their high moisture content. In order to mitigate the risk of microbial contamination and ensure physicochemical stability, it may be advisable to consider the implementation of a unitary drying operation. In this regard, the establishment of infrastructure and technological frameworks should be one of the main considerations of government policy, with the objective of promoting the effective utilization of food waste and by-products [2,218].

To fully harness the nutritional potential of plant-based oil industry by-products while ensuring safety and quality, it is essential to compare extraction methods by clearly outlining the bioactive products obtained, alongside their advantages and limitations, thereby supporting informed application and regulatory decisions (Table 8).

When formulating efficacious functional foods, one of the key considerations is the bioaccessibility and bioavailability of bioactive compounds within the human body after consumption. For instance, the bioavailability of many phenolic compounds is limited due to their low water solubility, chemical instability, interaction with the food matrix, and other nutrients [219]. Additionally, the bioaccessibility and bioavailability of bioactive compounds are highly affected by food processing conditions such as time and temperature. Therefore, further in vitro and in vivo studies are required to improve our understanding of the gastrointestinal fate of bioactive compounds within complex food matrices at scientific and industrial levels.

Another important criterion for formulating new food products with oil industry waste/by-products is the sensory quality of the final products and consumer acceptance. Collaborating with food companies on consumer testing can lead to improvements in the quality of fortified products, the design of new food formulations, and an understanding of consumer expectations. A significant number of studies have been published in the academic literature on the subject of the valorization of oil industry waste and by-products as natural antioxidants in oils. Hence, it can be highly advantageous to develop new product formulations by combining the waste/by-products with the products produced in the same facility on a large scale. This approach has the potential to yield favorable outcomes in terms of food safety and financial considerations. At present, the range of products available at the industrial level is extremely limited; thus, there is a need for further investigation and governmental support at the industrial level for companies with a view to reducing environmental problems, meeting consumer demands, and benefiting from low-cost and high-quality food waste/by-products as new food ingredients.

From a processing perspective, incorporating innovative technologies (e.g., green extraction processes) into industrial production offers significant potential for improving energy efficiency, reducing environmental pollution, and supporting sustainable development. These improvements can enhance environmental quality, minimize toxicity, promote human safety, and generate economic benefits. However, more information is needed to optimize processing conditions, since the efficiency of extraction and the quality of the final product depend on processing parameters and individual characteristics of raw materials. Furthermore, additional life cycle analysis data are required to ascertain the environmental and economic impact of different methods of procuring bioactive compounds. Therefore, cooperation between the scientific, industrial, and governmental communities is necessary to install and adapt innovative green processing techniques in large-scale production systems, ensuring the flow of information and economic activity while protecting the environment and meeting consumer demand.

Finally, it is important to mention that there is limited research and patent activity in the utilization of food waste and by-products in the scientific field and industry due to a lack of legislation [220]. In Europe, for instance, the regulation of the production of bioactive compounds extracted from food waste/by-products is based on the general food law and particularly on European Community (EC) Regulation No. 178/2002, Article 2, and Codex Alimentarius guidelines [221]. In addition, Commission Regulation (EC) No 1881/2006, which establishes maximum levels for certain contaminants in foodstuffs, does not encompass by-products such as pomace, peels, skins, spent grains, whey, or oilseed cakes and meals. Thus, by-products of food processing are not sufficiently taken into account by the current food safety legislation [222]. This situation is an urgent indicator that there is a need for effective improvements to policies, guidelines, rules, and codes that govern the safety limits and utilization of bioactive compounds derived from food waste in food products, dietary supplements, and pharmaceuticals.

## 5. Conclusions

The oil production from plant-based sources generates huge amounts of waste/by-products that are rich in nutritious, bioactive, and health-promoting compounds. Thus, these waste/by-products offer significant potential for incorporation into food systems as functional ingredients that can enhance nutritional value, improve health outcomes, and meet the growing demand for sustainable food production and clean-label food products. Novel and innovative technologies have made the recovery and utilization of these compounds increasingly viable. Valorizing such waste/by-products provides a potential contribution to reducing agricultural processing waste and promoting environmental sustainability. Furthermore, developing value-added food products can create economic opportunities. Despite current challenges relating to the standardization, safety, and regulatory approval of utilizing plant-based oil production waste and by-products, ongoing research and technological advancements continue to support integrating these bioactive-rich materials into the food industry. Consequently, the strategic use of plant-based oil processing waste/by-products could engender a circular economy, improve public health, and facilitate the transition to more sustainable food systems.

## Figures and Tables

**Table 1:** Application of olive oil waste/by-products as natural food additives in different food products.

Waste/By-Products	Form of the Additive	Food Product	The Final Quality of the Fortified Product	References
Olive pomace	Flour: 5 and 10% flour substitution	Pasta	↑ TPC: 2.28 and 0.69 mg GAE/g dw for uncooked and cooked, respectively ↑ Antioxidant potential (DPPH and ABTS) ↓ Rapidly digestible starch and ↑ slow digestible starch and resistant starch	[56]
Olive pomace	Fermented (*Saccharomyces cerevisiae*) flour: 20% flour substitution	“Taralli” (bakery products)	↑ TPC: up to 1377 μg/g dw (mainly hydroxytyrosol, tyrosol, verbascoside, oleacin, oleocanthal) ↑ Polyphenols (hydroxytyrosol, verbascoside, oleacin), triterpenic acids, tocochromanols (*α*-tocopherol, *β*-tocotrienol), carotenoids (mainly lutein) Storage at 25 °C for 90 days: ≈ bioactive compounds	[60]
Olive mill wastewater	Phenolic extract: 100 ppm	Sunflower oil and rapeseed oil	↓ Oxidative deterioration: acid value, PV, and extinction coefficient (K_270_) Accelerated storage at 60 °C for 4 weeks: ↓ the loss of phytosterols and tocopherols	[61]
Olive mill solid waste	Encapsulated phenolic extract: 100 mg/100 g	White soft cheese	↑ TPC and antioxidant capacity (DPPH) ↑ Total solids and protein Storage at 5 °C for 30 days: ≈ antioxidant capacity	[62]
Olive leaves	Phenolic extract: 75 g in 250 g flour	“Taralli” (bakery product)	↑ TPC, TFC, and antioxidant capacity (FRAP) for uncooked and cooked pasta and after in vitro digestion	[63]
Olive leaves and olive mill wastewater	Phenolic extract: 500 and 1000 mg/kg	Gluten-free breadstick	↑ TPC (mg GAE/100 g): from (control) 162.87 up to 139.68 with olive leave extract and 130.64 with olive mill wastewater extract ↑ Antioxidant activity (DPPH) ↑ Soluble and ↓ insoluble polyphenol fraction ↑ Bioaccessibility of polyphenols: up to 23.0 and 15.1% for fortification with olive leaves and olive mill wastewater, respectively ↑ Induction period via Oxitest	[64]
Olive pomace	Phenolic extract conjugate with inulin: 50 and 100 mg/mL	Pear beverage	↑ TPC: up to 28.59 GAE/L ↑ Antioxidant potential (DPPH and ABTS) Storage at 4 °C for 20 days: ≈ TPC, and antioxidant potential	[65]
Olive pomace	Flour: 10 and 20% flour substitution	Biscuit	↑ Dietary fiber, mineral, and lipid contents ↓ Total carbohydrates ↓ Better results with 10% addition	[66]

↑: Upregulation, ↓: downregulation, ≈: no statistical difference. ABTS: 2,2′-azino-bis(3-ethylbenzothiazoline-6-sulfonic acid) assay, DPPH: 2,2-diphenyl-1-picrylhydrazyl assay, dw: dry weight, FRAP: ferric reducing antioxidant power assay, GAE: gallic acid equivalent, TFC: total flavonoid content, TPC: total phenolic content.

**Table 2:** Application of avocado oil waste/by-products as natural food additives in different food products.

Waste/By-Products	Form of the Additive	Food Product	The Final Quality of the Fortified Product	References
Avocado seed	Flour: 6, 12, and 18%	Cereal snacks	↑ TPC: up to 167.78 mg GAE/100 g dw ↑ Antioxidant capacity (ABTS): up to 14.62 mmol TE/100 g dw ↑ Fiber content and ↓ carbohydrate and protein content	[117]
Avocado peel	Phenolic extract: 0.5% and 1%	Beef and soy-based burgers	↑ Protein, fat, and ash contents During cooking and storage: ↓ TBARS value, hexanal, and carbonyl content ↓ Heterocyclic aromatic amines and acrylamide after cooking	[118]
Avocado peel	Phenolic extract: 0.5% and 1.0%	Mayonnaise	Antimicrobial on *Escherichia coli* and *Staphylococcus aureus* ≈ Emulsion stability, and free fatty acids Refrigerated storage for 6 weeks: ↓ PV, *p*-AV, TOTOX value	[119]
Avocado seed	Flour: 5%, 10%, 15%, and 20%	*Injera* (fermented food)	↑ TPC: up to 265.76 mgGAE/100 g ↑ TFC: up to 72.37 mg QE/100 g ↑ Antioxidant potential (DPPH, FRAP, ABTS) ↑ Vitamin C and vitamin A: up to 8.28 and 26.01 mg/100 g, respectively ↑ *β*-carotene: up to 155.74 μg/100 g ↓ Anti-nutritional factor: phytic acid ↑ Protein, fat, and fiber contents and ↓ total carbohydrate content ↓ Rapidly digestible starch, and slowly digestible starch and ↑ resistant starch	[120]
Avocado pulp, seed, and peel	Flour: 5% and 10%	Semolina sourdough bread	↑ TPC: up to 23.882 mgGAE/g ↑ Antioxidant potential (DPPH assay: 9.234 mmol TE/100 g and ABTS: up to 6.656 mmol TE/100 g)	[121]
Avocado peel	Phenolic extract: keratin-starch composites functionalized with 0.0, 0.2, 0.6, and 1.0 mL extract	Freshly cut beef	↓ TBARS value, carbonyl content, and metmyoglobin level Storage at 4° for 12 days: ↓ yeast and mold count	[122]

↑: Upregulation, ↓: downregulation, ≈: no statistical difference. ABTS: 2,2′-azino-bis(3-ethylbenzothiazoline-6-sulfonic acid) assay, DPPH: 2,2-diphenyl-1-picrylhydrazyl assay, dw: dry weight, FRAP: ferric reducing antioxidant power assay, GAE: gallic acid equivalent, PV: peroxide value, *p*-AV: *p*-anisidine value, TBARS: thiobarbituric acid reactive substances, TE: Trolox equivalent, TFC: total flavonoid content, TPC: total phenolic content, TOTOX index: a total oxidation index.

**Table 3:** Application of coconut oil waste/by-products as natural food additives in different food products.

Waste/By-Products	Form of the Additive	Food Product	The Final Quality of the Fortified Product	References
Defatted coconut flour	Flour: 10, 20, 30, 40, and 50% flour substitution	Nixtamalized maize flour	↑ TPC: up to 116.40 mg GAE/100 g ↑ TFC: up to 94.81 mg/100 g ↑ Antioxidant potential (DPPH and FRAP assays) ↑ Protein and fat contents and ↓ carbohydrate content ↑ Dietary fiber content: up to 45.95 and 5.12% for insoluble and soluble dietary fiber, respectively	[154]
Coconut residue	Flour: 5, 10, 15, 20, and 25% flour substitution	Pasta	↑ Protein, fat, and total dietary fiber contents and ↓ carbohydrate content	[155]
Virgin coconut meal	Flour: 5, 10, 15, and 20% flour substitution	Cake	↑ Moisture, fat, protein, and ash contents, and ↓ carbohydrate content	[156]
Defatted coconut flour	Flour: 10, 20, 30, 40, and 50% flour substitution	Rice noodles	↑ TPC: up to 2.35 g/1000 g ↑ TFC: up to 4.62 g/1000 g ↑ Antioxidant activity: up to 14.71% for DPPH assay ↑ Mineral content: up to 21.40, 326.47, 9.17, 0.36, 1.15, and 1.26 ppm for Na, K, Ca, Cu, Fe, and Zn	[157]
Virgin coconut oil cake	Flour: 10, 20, 30, 40 and 50% flour substitution	Muffin	↑ Protein, fat, crude fiber, and total mineral contents Storage at 4 °C and 35 °C for 16 days: no change in the quality	[158]
Virgin coconut oil cake	Flour: 20, 25, 30% flour substitution	Extruded snacks	The optimized conditions: 28.7% virgin oil cake, 14% feed moisture, and 300 rpm screw speed Optimized products: moisture, carbohydrate, protein, fat, ash, and crude fiber content: 5.1, 74.19, 11.14, 5.07, 2.3, and 1.58 g/100 g	[159]

↑: Upregulation, ↓: downregulation. DPPH: 2,2-diphenyl-1-picrylhydrazyl assay, dw: dry weight, GAE: gallic acid equivalent, TFC: total flavonoid content, TPC: total phenolic content, TOTOX index: a total oxidation index.

**Table 4:** Application of rapeseed/canola oil waste/by-products as natural food additives in different food products.

Waste/By-Products	Form of the Additive	Food Product	The Final Quality of the Fortified Product	References
Rapeseed meal	Phenolic-rich washout: 5, 15%	Soybean, rapeseed, and sunflower oil	↑ Induction period by Rancimat method Comparable antioxidant activity index at 15% level to BHT (0.02%)	[168]
Canola meal	Nano-encapsulated phenolic extract: 200, 400, and 800 ppm	Canola oil, storage at 30 °C for 60 days	↓ PV, TBARS level ↑ Antioxidant activity (DPPH), iodine value, ↑ Induction period by Rancimat method: highest at 800 ppm: 12.9 h Comparable to TBHQ at 200 ppm; superior effect at 400–800 ppm	[169]
Rapeseed meal	Acid-hydrolyzed + lyophilized Extract: 200 ppm	Oil collected after 24 h of deep-frying French fries	↓ PV, *p*-AV, K232, K268 values ↓ TOTOX index: 221.10 (control) →196.8 (with extract) ↓ INTOX index: 390.72 (control) → 343.16 (with extract) ↑ TPC and antioxidant potential (ABTS, FRAP, DPPH)	[170]
Rapeseed cake, with/without black cumin cake	Flour: 1:6 ratio	Plant-based beverage, microwave pre-treated	↑ TPC, tocopherol, carotenoid, and chlorophyll content Optimum conditions: 800 W, 3 sec, black cumin/rapeseed meal ratio: 0.1144	[171]
Rapeseed press cake	Flour: 20, 40% flour substitution	Biscuit	Antioxidant activity: from 535–80 to 8375–10 088 µmol TE/100 g Optimized formula: 40% rapeseed cake + 2.3% saturated fat	[172]

↑: Upregulation, ↓: downregulation. ABTS: 2,2′-azino-bis(3-ethylbenzothiazoline-6-sulfonic acid), BHT: butylated hydroxytoluene, DPPH: 2,2-diphenyl-1-picrylhydrazyl, FRAP: ferric reducing antioxidant power, INTOX index: integral oxidation index, K_232_ and K_268_: specific extinction values, PV: peroxide value, *p*-AV: *p*-anisidine value, TBARS: thiobarbituric acid reactive substances, TBHQ: *tert*-butylhydroquinone, TE: Trolox equivalent, TFC: total flavonoid content, TPC: total phenolic content, TOTOX index: a total oxidation index.

**Table 5:** Application of sunflower oil waste/by-products as natural food additives in different food products.

Waste/By-Products	Form of the Additive	Food Product	The Final Quality of the Fortified Product	References
Sunflower meal	Phenolic-rich washout: 5, 15%	Soybean, rapeseed, and sunflower oil	↑ Induction period by Rancimat method Comparable antioxidant activity index at both levels to BHT (0.02%)	[168]
Dehulled sunflower press-cake	Protein concentrate: 10%	Protein-rich sport beverages	↑ TPC compared to whey and pea protein concentrate ↓ Total phenolic bioaccessibility: 84.3% reduction	[179]
Sunflower meal	Phenolic-rich washout: 10, 20, 30, 40, and 50%	Whey protein dispersion	↑ Protein content ↑ TPC ↓ Available amino groups	[180]
Sunflower pomace	Phenolic extract: 5, 10, and 20%	Strawberry puree Strawberry + yogurt beverage	↑ Antioxidant potential (CUPRAC, ABTS, DPPH) before and after digestion	[16]
Sunflower seed cake	Flour: 5, 10, 15% flour substitution	Gluten-free bread	↑ Protein, crude fiber, fat TPC: 89.30 to 222.33 mg GAE/100 g	[181]
Sunflower meal	Flour: 2, and 4%	Frankfurter	↑ Protein and dietary fibre ↑ Mineral content: Mg, K, Fe, Zn, Cu, Mn ↑ TPC from 51 to 76 and 93 mg GAE/100 g, respectively.	[182]

↑: Upregulation, ↓: downregulation. ABTS: 2,2′-azino-bis(3-ethylbenzothiazoline-6-sulfonic acid) assay, BHT: butylated hydroxytoluene, CUPRAC: cupric reducing antioxidant capacity, DPPH: 2,2-diphenyl-1-picrylhydrazyl assay, GAE: gallic acid equivalent, TPC: total phenolic content.

**Table 6:** Application of flaxseed oil waste/by-products as natural food additives in different food products.

Waste/By-Products	Form of the Ingredient	Food Product	The Final Quality of the Fortified Product	References
Flaxseed press cake	Flour: 4 and 8% flour substitution	Bread	↑ Antioxidant potential (DPPH, ABTS and FRAP) ↓ Glucose release after in vitro digestion	[192]
Flaxseed marc	Flour: 5, 15, 25% flour substitution	Bread	↑ Antioxidant potential (DPPH: from (control) 19.50% to 54.25%)	[187]
Flaxseed cake	Flour: 5, 7.5, and 10% flour substitution	Bread, sourdough bread	↑ TPC and TFC ↑Antioxidant potential (DPPH, TEAC)	[198]
Flaxseed cake	Flour: 5, 7.5, and 10% flour substitution	Sourdough bread	↑ Total phenolics and flavonoids ↑Antioxidant potential (DPPH, ABTS)	[199]
Flaxseed cake	Hot-distilled water extract: 25, 50, 75, and 100%	Gluten-free bread	↑ Protein, ↓ carbohydrates ↑ Mineral content: P, K, Mg ↑ TPC: from (control) 0.096 to 0.234 mg GAE/g ↑ Antioxidant potential (DPPH, ABTS, FRAP)	[200]
Flaxseed meal	Flour: 5, 10, 15% flour substitution	Toast and cake	↑ Mineral content: K, Mg, P, and Ca	[186]
Flaxseed cake	5%, 10%, and 15% in water	Kefir-like fermented beverage with kefir grains, storage for 21 days at 6 °C	↑ TPC, TFC, and antioxidant potential (DPPH, ABTS): linear with flaxseed ratio Stable phenolic and antioxidant potential during storage	[201]
Flaxseed cake	35% in water	Camembert analog with *Penicillium camemberti* (PC) alone or with yeast *Geotrichum candidum*,	Storage for 28 days: ↑ TPC and TFC Antioxidant potential: ↑ 0–7/14 days ↓ 21–28 days	[202]
Flaxseed cake	1.5 and 3%	Mortadella	↓ TBARS level	[188]

↑: Upregulation, ↓: downregulation. ABTS: 2,2′-azino-bis(3-ethylbenzothiazoline-6-sulfonic acid) assay, BHT: butylated hydroxytoluene, DPPH: 2,2-diphenyl-1-picrylhydrazyl assay, FRAP: ferric reducing antioxidant power, TFC: total flavonoid content, TPC: total phenolic content.

**Table 7:** Application of sesame seed oil waste/by-products as natural food additives in different food products.

Waste/By-Products	Form of the Additive	Food Product	The Final Quality of the Fortified Product	References
Sesame seed cake	Flour: 5, 5.15, 6, 8, and 10% flour substitution	Mayonnaise	↓ PV, *p*-AV	[205]
Sesame seed meal	Soaked and filtered milk-like suspension: 6, 7, and 8% flour in water	Yogurt	↑ Antioxidant potential (DPPH, FRAP, ABTS, ORAC)	[213]
Sesame seed cake	A filling mixture of date paste and sesame seed cake flour (80:20)	Biscuit	↑ Protein, fiber, and ↓ reducing sugar, total sugar ↑ TPC and TFC, antioxidant activity (DPPH) ↑ Mineral content: Ca, Zn, Fe, Mg	[207]
Sesame seed cake	Flour: 20, 30, and 40% flour substitution	Biscuit	↑ Protein, fiber, and ↓ carbohydrates ↑ Mineral content: P, Mg, Ca	[206]
Sesame seed cake	Flour: 5, 10, 20% flour substitution	Cookie	↑ TPC, antioxidant potential (FRAP, TEAC) before and after digestion	[209]
Sesame seed meal	Flour	Cake	↑ Protein, fiber, and ↓ carbohydrates ↑ Mineral content: Ca, Zn, Fe	[214]
Sesame seed cake	Flour: 6, 12, and 20% flour substitution	Bread	↑ TPC, total lignans, and antioxidant potential (DPPH, ABTS) ↑ Caffeic acid, ferulic acid, sinapic acid, and *p*-coumaric acid, sesaminol diglucosides and sesaminol triglucosides	[215]
Sesame seed cake	Flour: 20, 40, and 60% soy protein isolate substitution	High-moisture extrusion-processed meat analogs	↑ Free hydroxybenzoic acids ↓ Free and ↑ bound vitexin ↑ Free and bound quercetin, trans-cinnamic acid New flavonoids: naringenin, luteolin, and apigenin	[208]

↑: Upregulation, ↓: downregulation. ABTS: 2,2′-azino-bis(3-ethylbenzothiazoline-6-sulfonic acid) assay, DPPH: 2,2-diphenyl-1-picrylhydrazyl assay, FRAP: ferric reducing antioxidant power, ORAC: oxygen radical absorbance capacity, PV: peroxide value, *p*-AV: *p*-anisidine value, TFC: total flavonoid content, TPC: total phenolic content.

**Table 8:** Comparison of extraction methods for plant-based oil Industry waste/by-products.

Waste/By-Product	Extraction Method	Products	Advantages	Disadvantages
Olive pomace	Solid–liquid extraction	Phenolics, fiber, proteins, fatty acids	Simple, widely used	Low yield, long time
	Ultrasound-assisted	Phenolics, antioxidants	Faster, higher yield	Requires special equipment
	Pressurized liquid extraction	Phenolics, flavonoids	High phenolic content, fast	Thermal degradation risk
	NADES	Phenolics, flavonoids	Green, selective	Needs optimization
	Methanol maceration	Polyphenols, flavonoids	High efficiency	Toxic solvent
	Microwave-assisted	Phenolics	Fast, energy-efficient	Equipment cost
Olive leaves	Ultrasound-alkaline-assisted extraction	Proteins, bioactive peptides	Mild conditions, preserves activity	Enzyme cost
	Enzyme-assisted extraction	Proteins	Effective protein extraction	May denature some proteins
Palm kernel cake	Alkaline solubilization	Proteins, bioactive peptides	Effective protein extraction	Possible protein denaturation
	Enzyme hydrolysis	Antioxidant peptides	Functional peptides produced	Enzyme cost, control needed
	Microwave-assisted	Phenolics	High yield, fast extraction	Equipment cost
	Ultrasound-assisted	Phenolics, sterols, carotenoids	Enhanced recovery	Equipment needed
	NADES	Phenolics	Green, selective	Emerging method, optimization needed
Avocado by-products	Mechanical, chemical, microwave, ultrasound, DES	Phenolics, carotenoids, proteins, lipids	Rich bioactives, green methods	Variability, limited protein data
Coconut by-products	Various (e.g., copra meal, press cake, fiber)	Proteins, fibers, phenolics	Nutritional value, functional properties	Some variability
Rapeseed meal	Alkaline extraction	Protein isolates	High yield, low cost	Protein denaturation, low solubility
	NADES extraction	Enhanced protein	Green, selective, high solubility	High viscosity, limited scalability
	Aqueous ethanol extraction	Phenolic compounds	Food-grade, effective for free phenolics	Less effective for bound phenolics
Sunflower meal	Aqueous extraction	Phenolics (e.g., chlorogenic acid)	Simple, cost-effective	Co-extraction of unwanted pigments
	Dehulling + pressing	Protein-rich meal	Higher protein and phenolic content	Requires additional preprocessing
Flaxseed cake	Steam explosion	Bound phenolics, lignans	High release efficiency	Energy-intensive
	Ultrasound-assisted hydrolysis	Antioxidant peptides	Improved bioactivity and yield	Equipment cost
	Microwave treatment	Phenolics, minerals	Enhances extractability	Risk of thermal degradation
Sesame seed cake	Enzymatic hydrolysis	Bioactive peptides	Strong antioxidant potential	Enzyme cost, optimization needed
	NADES extraction	High-purity proteins	Eco-friendly, high solubility	Still limited at industrial scale
	Organic solvent extraction	Lignans, flavonoids	Effective and widely used	Solvent toxicity, environmental concerns

## Data Availability

No new data were created or analyzed in this study. Data sharing does not apply to this article.

## Abbreviations

The following abbreviations are used in this manuscript:

ABTS	2,2′-azino-bis(3-ethylbenzothiazoline-6-sulfonic acid) assay
BHA	Butylated hydroxyanisole
BHT	Butylated hydroxytoluene
CAGR	Compound annual growth rate
DPPH	2,2-diphenyl-1-picrylhydrazyl assay
dw	Dry weight
FAO	Food and Drug Organisation
FRAP	Ferric reducing antioxidant power assay
GAE	Gallic acid equivalent
HPMC	Hydroxypropyl methylcellulose
ELISA	Enzyme-linked immunosorbent assay
NADES	Natural deep eutectic Ssolvents
TBARS	Thiobarbituric acid-reactive substances
TBHQ	Tert-butylhydroquinone
